# Latent Class Analysis to Identify Novel Phenotypes in Exacerbations of COPD: A Retrospective, Multicenter Cohort Study

**DOI:** 10.1002/mco2.70444

**Published:** 2025-10-28

**Authors:** Xiangqing Hou, Zhishan Deng, Yumin Zhou, Jie Hong, Fan Wu, Yuemao Li, Jinrong Huang, Cuiqiong Dai, Lifei Lu, Gaoying Tang, Qi Wan, Kunning Zhou, Xiaohui Wu, Jieqi Peng, Leqing Zhu, Ximo Chen, Pixin Ran

**Affiliations:** ^1^ Guangzhou National Laboratory Guangzhou International Bio Island Guangzhou China; ^2^ State Key Laboratory of Respiratory Disease National Clinical Research Center for Respiratory Disease Guangzhou Institute of Respiratory Health The First Affiliated Hospital of Guangzhou Medical University Guangzhou Medical University Guangzhou China; ^3^ Information and Data Management Center Guangzhou Medical University Guangzhou China

**Keywords:** ECOPD, latent class analysis, multicenter study, phenotypes, personalized medicine

## Abstract

This study aimed to identify novel phenotypes in patients with exacerbations of chronic obstructive pulmonary disease (ECOPD) to enable precise management, as current phenotypic classifications show limited utility in predicting patient prognosis. By analyzing data from a robust, retrospective multicenter registry (*n* = 13,449) and leveraging 133 biomarkers with penalized Cox models, we developed a six‐phenotype latent class analysis model. Phenotype 1 is distinguished by elevated direct bilirubin (Dbil) and lactate dehydrogenase (LDH). Phenotype 2 features a higher percentage of lymphocytes (LYMPH_pct) and lower percentage of neutrophils (NEUT_pct). Phenotype 3 is marked by increased generalized cardiovascular disease (gCVD) and reduced NEUT_pct. Phenotype 4 is related to higher NEUT_pct and lower LYMPH_pct. Phenotype 5 is associated with a higher prevalence of gCVD and surgical trauma history. Phenotype 6 stands out for its higher rates of respiratory failure and elevated pulse at admission. Compared with Phenotype 1, patients in Phenotype 6 have a significantly higher risk of all‐cause mortality in both the development and validation sets, with adjusted hazard ratios of 2.06 (95% CI: 1.38–3.08) and 2.51 (95% CI: 1.43–4.04), respectively. These findings reveal novel ECOPD subgroups with significant prognostic differences, providing a crucial framework for implementing precision health management and improving patient outcomes.

## Graphical abstract

•This is the first study to identify six phenotypes with specific characteristics using 12 biomarkers from a large‐scale, multicentre cohort of patients with ECOPD.

•We have identified six different subgroups of patients with ECOPD using an unsupervised latent class analysis model, which were associated with different patterns of clinical manifestations.

•We found that compared with patients in Phenotype 1, those in Phenotype 6 had a higher risk of death perhaps attributed to high prevalence of respiratory failure, pulmonary heart disease, and generalized cardiovascular disease.

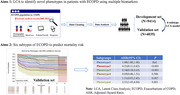

## 1 Introduction

The Global Burden of Disease Data 2021 reported that chronic obstructive pulmonary disease (COPD) is the third leading cause of death worldwide, and the precise management and control of COPD is a major public health issue [1]. Exacerbations of COPD (ECOPD) are significant events that contribute to disease progression and can even progress to death as well as increased healthcare burden. It has been reported that more than 50% of patients with ECOPD were unrecognized [2], and each exacerbation will increase the risk of recurrence and shorten the time interval to the next episode, which is associated with poor prognosis and several serious comorbidities.

Traditionally, ECOPD has been classified based on clinical features and severity, but there is growing recognition that COPD is a heterogeneous disease with various underlying phenotypes; therefore, the clinical and pathological phenotypes of different patients with ECOPD vary widely. In response to the heterogeneity and complexity of COPD and to achieve precise intervention, Agusti et al. [3] proposed the “Treatable Traits” strategy, which aims to predict the risk of ECOPD by identifying patient‐specific phenotypes or biomarkers that have been verified for causality and provide a more precise treatment. However, there is still a lack of well‐established biomarkers to predict ECOPD prognosis in clinical practice. Perhaps the reasons are, to our knowledge, the lack of biomarkers with strong clinical evidence associated with the occurrence of ECOPD, which has been verified by large‐scale, prospective population cohorts. Moreover, the effective integration of massive clinical datasets remains a challenge, and traditional statistical methods are often incapable of fully leveraging the information contained in multi‐dimensional clinical samples. Therefore, there is currently a lack of accurate disease classification of ECOPD to predict progression outcomes, which poses a huge challenge for early screening and treatment of COPD.

Phenotyping aims to classify COPD patients into distinct subgroups based on unique disease attributes. These subgroups share specific prognostic or therapeutic characteristics, helping to predict their risk of clinically important outcomes like exacerbations or mortality [4]. The “Rome classification” recommends that clinicians and researchers use six objectively measured variables (dyspnea, respiratory rate, heart rate, oxygen saturation, and C‐reactive protein and, in selected cases, arterial blood gases) to assess the severity of ECOPD [5]. However, whether the Rome criteria can be effectively applied to evaluate ECOPD severity in real‐world cohorts of hospitalized patients remain under debate [6]. Additionally, prior to diagnosing ECOPD, careful attention should be given to the differential diagnosis of conditions with similar clinical manifestations, such as heart failure, pneumonia, and pulmonary thromboembolism. Many patients with ECOPD also have chronic comorbidities, including ischemic heart disease and heart failure, which may develop or worsen during the course of the disease [7]. A prospective population‐based study further indicated that the number and type of COPD comorbidities are associated with an increased risk of future mortality [8].

Latent class analysis (LCA) is an unsupervised machine learning method. It allows clustering categorical variables and stratifies individuals into distinct clusters based on responses to selected variables [9]. Recently, LCA has been widely used to identify homogeneous subgroups within populations. It has significantly improved the accuracy of disease prediction and early intervention by identifying novel disease phenotypes [10, 11]. Studies have also indicated that LCA can effectively capture the heterogeneity of COPD [12, 13]. However, the reproducibility of these subtypes remains a major challenge [14]. Research focusing on the application of LCA in ECOPD is still very limited.

This study aimed to uncover new clinical phenotypes within patients experiencing ECOPD to better stratify their risks of all‐cause mortality and recurrence. We successfully identified and validated six distinct phenotypes with significantly different prognoses. These findings are important as they provide a framework for individualized care, helping clinicians move beyond uniform approaches and tailor interventions for the most vulnerable patients with ECOPD.

## 2 Results

### 2.1 Study Population Characteristics

In total, we obtained 68,509 medical records of COPD patients in this study. Finally, 13,449 patients with ECOPD met the inclusion criteria and were included in the analysis (Table S1). These patients contributed to 26,284 medical records in the longitudinal electronic medical records (EMRs), and the recurrence rate was 33.8% in this study. The ratio of man to woman is 5:1 and the median (P_25_, P_75_) age of study population is 73 (65, 80) years. The age distribution of the study population is presented in Figure S1. Since the majority of individuals (99.9%) were over 40 years old and no established cutoff value exists to define elderly ECOPD patients, we used the median age of our cohort (73 years) as the threshold for subsequent analyses. Most of the patients (64.4%) come from the respiratory department, while 2010 (15.0%) patients were derived from the intensive care unit (ICU). The median length of stay for ECOPD patients was 8 days, with an interquartile range of 6–11 days.

### 2.2 Feature Selection and Model Construction

**FIGURE 1:**
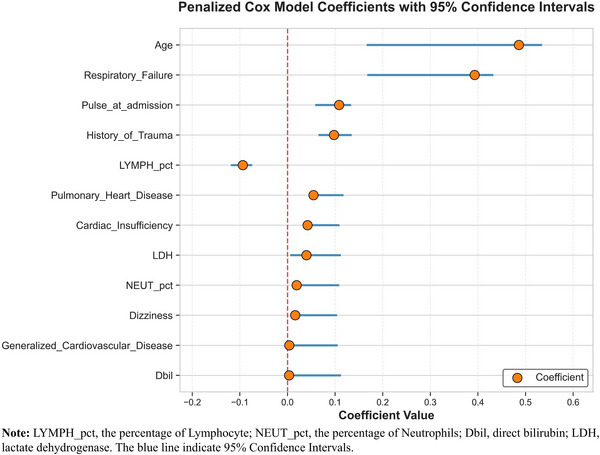
Forest plot of penalized cox proportional hazards model coefficients with 95% confidence intervals. This figure presents the estimated coefficients and 95% confidence intervals (blue lines) for the top 12 most influential features identified by an elastic‐net‐regularized Cox survival analysis model. A footnote is provided that lists all variable acronyms alongside their full expanded forms.

All participants were randomly divided into development and validation datasets at a ratio of 7:3. The feature selection and model construction are depicted in Figure S2. Based on the development dataset, elastic‐net penalized Cox models were first used to obtain 12 features from 66 features for clustering analysis (Figure 1), including respiratory failure, history of surgical trauma, pulmonary heart disease, cardiac insufficiency, dizziness, generalized cardiovascular disease (gCVD), age, pulse at admission, the percentage of lymphocytes (LYMPH_pct), direct bilirubin (Dbil), lactate dehydrogenase (LDH), and the percentage of neutrophils (NEUT_pct).

Moreover, we fitted cubic spline curves to explore dose–response relationships of the five continuous variables with all‐cause mortality, and the results showed that Dbil, LYMPH_pct, and LDH exhibited an approximately linear relationship with mortality risk, while age and NEUT_pct showed a slightly non‐linear positive association with mortality (Figures S3–S7). Furthermore, no significant interaction was observed between age and NEUT_pct (*p* = 0.140), suggesting that our original model remained stable when these two variables were included. To further assess potential multicollinearity among the selected variables, we performed a multicollinearity test (Table S2). The variance inflation factors (VIFs) for all 12 predictors were below 5. This indicates that multicollinearity was not a substantial concern in the formal analysis.

For the purposes of LCA and enhanced clinical interpretation, the variables, including age, pulse at admission, LYMPH_pct, Dbil, LDH, and NEUT_pct, were dichotomized based on their median values. Based on the patients’ responses to the 12 categorical indicator variables, we derived a six‐phenotype model. This model can be used to identify ECOPD patients at higher risks of all‐cause mortality. Finally, a support vector machine (SVM) classifier was developed to predict phenotypes leveraging the 12 important features.

### 2.3 Comparison of Clinical Information Between Phenotypes

Ultimately, the six‐phenotype LCA model was selected as the optimal fit to identify patients with ECOPD at a higher risk of all‐cause mortality. The six‐phenotype pattern was revealed by LCA based on the lowest Akaike information criterion (AIC), Bayesian information criterion (BIC), the adjusted BIC using Rissanen's sample size adjustment (SSA‐BIC), and the “consistent AIC” (CAIC) criteria (Table S3). The results also support six‐phenotype LCA model superior than five‐class and seven‐class models because the bootstrap likelihood ratio test (BLRT) and Lo–Mendell–Rubin likelihood ratio (LMR) test results achieved significant (*p* < 0.001). Moreover, we additionally reported the fit statistics of the LCA model based on the multiple imputation dataset (Table S4). The results show that the LCA model with six classes has the lowest BIC, SSA‐BIC, and CAIC than other models. Although the LCA model with seven classes has a lower AIC than the six‐class model, the latter one has a higher entropy value than the former one (0.83 vs. 0.82). Additionally, according to Occam's Law of Razor [15], the superiority model for achieving optimal results is a model with fewer variables. Therefore, we are choosing the LCA model with six classes in this study as the optimal model, which further validates the good and robust performance of our LCA model. Furthermore, Table S4 suggests that the BLRT and LMR differed for the model consisting of seven classes, so preference was given to the significant BLRT (*p* < 0.001) and LMR (*p* = 0.050) of the six‐class model. Hence, the results also support that the LCA model with six classes is acceptable, and our findings are reliable.

Besides, the clinical interpretation was also considered in this study since the six‐phenotype LCA model had an obvious separation and distinct clinical characteristics. As shown in Table 1, Phenotype 1 is characterized by older age (≥ 73 years) and higher levels of Dbil, LDH, and NEUT_pct. Phenotype 2 is associated with higher LYMPH_pct and lower NEUT_pct, and in contrast, patients in Phenotype 4 have lower LYMPH_pct but higher NEUT_pct. Phenotype 3 is obviously related to a higher prevalence of gCVD and lower NEUT_pct. Phenotype 5 is characterized by higher rates of gCVD and older age as well as higher Dbil, LDH, and NEUT_pct. Phenotype 6 stands out for its higher rates of respiratory failure, pulmonary heart disease, gCVD, and elevated pulse at admission, LDH, NEUT_pct, as well as reduced LYMPH_pct. Moreover, the comparison of the baseline characteristics, comorbidities, clinical symptoms, medical history, blood, and exhaled breath biomarkers among various phenotypes is described in Table S5. Of note that 32.1% of patients in Phenotype 5 were came from ICU, which is significantly larger than other phenotypes (*p* < 0.001). Furthermore, significant differences were observed among the six groups regarding hospital source, medical insurance status, and whether inhaled corticosteroids were used for therapy. Therefore, the confounding effects of the above factors should be well considered when investigating the associations between the novel phenotypes and prognostic outcomes.

**TABLE 1:** The comparison for the 12 predictors among six subgroups in the development dataset.

Variables	Phenotype 1 *n* = 1104	Phenotype 2, *n* = 2873	Phenotype 3, *n* = 1589	Phenotype 4, *n* = 1525	Phenotype 5, *n* = 1566	Phenotype 6, *n* = 757	*p*
Respiratory failure							< 0.001
No	1005 (91.0)	2645 (92.1)	1244 (78.3)	1166 (76.5)	1251 (79.9)	295 (39.0)	
Yes	99 (9.0)	228 (7.9)	345 (21.7)	359 (23.5)	315 (20.1)	462 (61.0)	
History of surgical trauma							< 0.001
No	652 (59.1)	2420 (84.2)	1115 (70.2)	1462 (95.9)	577 (36.8)	734 (97.0)	
Yes	452 (40.9)	453 (15.8)	474 (29.8)	63 (4.1)	989 (63.2)	23 (3.0)	
Pulmonary heart disease							< 0.001
No	1104 (100.0)	2873 (100.0)	974 (61.3)	1525 (100.0)	1059 (67.6)	97 (12.8)	
Yes	0 (0.0)	0 (0.0)	615 (38.7)	0 (0.0)	507 (32.4)	660 (87.2)	
Cardiac insufficiency							< 0.001
No	1104 (100.0)	2873 (100.0)	1229 (77.3)	1525 (100.0)	878 (56.1)	683 (90.2)	
Yes	0 (0.0)	0 (0.0)	360 (22.7)	0 (0.0)	688 (43.9)	74 (9.8)	
Dizziness							< 0.001
No	639 (57.9)	2051 (71.4)	969 (61.0)	1143 (75.0)	721 (46.0)	589 (77.8)	
Yes	465 (42.1)	822 (28.6)	620 (39.0)	382 (25.0)	845 (54.0)	168 (22.2)	
Generalized cardiovascular disease							<0.001
No	1019 (92.3)	2778 (96.7)	0 (0.0)	1256 (82.4)	0 (0.0)	0 (0.0)	
Yes	85 (7.7)	95 (3.3)	1589 (100.0)	269 (17.6)	1566 (100.0)	757 (100.0)	
Age, years							< 0.001
< 73	0 (0.0)	2283 (79.5)	964 (60.7)	933 (61.2)	50 (3.2)	393 (51.9)	
≥ 73	1104 (100.0)	590 (20.5)	625 (39.3)	592 (38.8)	1516 (96.8)	364 (48.1)	
Pulse at admission							< 0.001
< 88	444 (40.2)	1134 (39.5)	603 (37.9)	320 (21.0)	636 (40.6)	116 (15.3)	
≥ 88	660 (59.8)	1739 (60.5)	986 (62.1)	1205 (79.0)	930 (59.4)	641 (84.7)	
LYMPH_pct							< 0.001
< 14.4	701 (63.5)	290 (10.1)	195 (12.3)	1524 (99.9)	1095 (69.9)	730 (96.4)	
≥ 14.4	403 (36.5)	2583 (89.9)	1394 (87.7)	1 (0.1)	471 (30.1)	27 (3.6)	
Dbil, µmol/L							<0.001
< 2.6	113 (10.2)	1972 (68.6)	821 (51.7)	1033 (67.7)	194 (12.4)	352 (46.5)	
≥ 2.6	991 (89.8)	901 (31.4)	768 (48.3)	492 (32.3)	1372 (87.6)	405 (53.5)	
LDH, U/L							< 0.001
< 189	260 (23.6)	2020 (70.3)	927 (58.3)	794 (52.1)	347 (22.2)	221 (29.2)	
≥ 189	844 (76.4)	853 (29.7)	662 (41.7)	731 (47.9)	1219 (77.8)	536 (70.8)	
NEUT_pct							< 0.001
< 76.1	93 (8.4)	2769 (96.4)	1589 (100.0)	95 (6.2)	18 (1.1)	97 (12.8)	
≥ 76.1	1011 (91.6)	104 (3.6)	0 (0.0)	1430 (93.8)	1548 (98.9)	660 (87.2)	

Abbreviations: Dbil, direct bilirubin; LDH, lactate dehydrogenase; LYMPH_pct, the percentage of lymphocytes; NEUT_pct, the percentage of neutrophils.

### 2.4 Association of Phenotypes With Clinical Outcomes

We evaluated the proportional hazards (PH) assumption for each Cox model presented in Table S6 using Schoenfeld residual tests. The results revealed violations of the PH assumption in both the univariate and multivariable Cox models. To address this issue, we performed an extended Cox regression analysis by incorporating time‐dependent interaction terms and stratifying covariates, ensuring more accurate hazard ratio (HR) estimates [16].

When controlling or stratifying for department original, hospital source, medical insurance types, medical history of COPD, and whether inhaled corticosteroids were used for therapy, patients in Phenotype 6 were observed to have a significantly higher risk of all‐cause mortality compared to those in Phenotype 1 in both the development and validation datasets (Table 2). In contrast, patients in Phenotype 2 exhibited the lowest adjusted HR (AHR) among all phenotypes. Moreover, Phenotype 6 was associated with the highest risk of ECOPD recurrence in the validation dataset. Furthermore, the adjusted time ratios (TR) derived from the Lognormal models (Table S7) also confirmed that Phenotype 6 was associated with significantly shorter survival time compared to Phenotype 1 (*p* < 0.05) in the development (TR = 0.26) and validation sets (TR = 0.08), respectively, indicating a poorer prognosis.

**TABLE 2:** Prognostic outcomes of ECOPD by phenotypes.

Outcomes	*N*	Cases (%)	HR (95% CI)	*p*	AHR (95% CI)	*p*
**Development**						
ECOPD recurrence						
Phenotype 1	1104	445 (40.3)	1.00 (1.00, 1.00)	Reference	1.00 (1.00,1.00)	Reference
Phenotype 2	2873	784 (27.3)	0.61 (0.54, 0.69)	< 0.001	0.69 (0.61, 0.79)	< 0.001
Phenotype 3	1589	509 (32.0)	0.79 (0.69, 0.91)	< 0.001	0.84 (0.73, 0.97)	0.015
Phenotype 4	1525	462 (30.3)	0.75 (0.65, 0.87)	< 0.001	0.86 (0.74, 1.01)	0.062
Phenotype 5	1566	591 (37.7)	1.13 (0.97, 1.31)	0.122	1.08 (0.93, 1.26)	0.309
Phenotype 6	757	221 (29.2)	0.81 (0.66, 0.98)	0.031	0.92 (0.76, 1.12)	0.418
Mortality						
Phenotype 1	1104	82 (7.4)	1.00 (1.00, 1.00)	Reference	1.00 (1.00, 1.00)	Reference
Phenotype 2	2873	33 (1.1)	0.17 (0.11, 0.26)	< 0.001	0.24 (0.16, 0.36)	< 0.001
Phenotype 3	1589	64 (4.0)	0.66 (0.47, 0.94)	0.020	0.74 (0.52, 1.05)	0.089
Phenotype 4	1525	48 (3.1)	0.55 (0.37, 0.80)	0.002	0.74 (0.50, 1.11)	0.147
Phenotype 5	1566	178 (11.4)	2.21 (1.62, 3.02)	< 0.001	1.80 (1.32, 2.47)	< 0.001
Phenotype 6	757	59 (7.8)	1.55 (1.06, 2.28)	0.025	2.06 (1.38, 3.08)	< 0.001
**Validation**						
ECOPD recurrence						
Phenotype 1	453	174 (38.4)	1.00 (1.00, 1.00)	Reference	1.00 (1.00, 1.00)	Reference
Phenotype 2	1200	360 (30.0)	0.77 (0.64, 0.92)	0.005	1.17 (0.93, 1.47)	0.179
Phenotype 3	756	229 (30.3)	0.87 (0.70, 1.07)	0.184	1.36 (1.01, 1.84)	0.047
Phenotype 4	633	184 (29.1)	0.83 (0.66, 1.06)	0.133	1.37 (1.07, 1.76)	0.014
Phenotype 5	678	243 (35.8)	1.30 (1.02, 1.66)	0.032	1.56 (1.18, 2.07)	0.002
Phenotype 6	315	96 (30.5)	1.12 (0.83, 1.52)	0.454	1.74 (1.23, 2.46)	0.002
Mortality						
Phenotype 1	453	32 (7.1)	1.00 (1.00, 1.00)	Reference	1.00 (1.00, 1.00)	Reference
Phenotype 2	1200	12 (1.0)	0.15 (0.08, 0.29)	< 0.001	0.21 (0.11, 0.42)	< 0.001
Phenotype 3	756	16 (2.1)	0.33 (0.18, 0.62)	< 0.001	0.34 (0.18, 0.63)	< 0.001
Phenotype 4	633	27 (4.3)	0.69 (0.40, 1.19)	0.179	0.85 (0.48, 1.51)	0.586
Phenotype 5	678	68 (10.0)	1.75 (1.09, 2.83)	0.021	1.51 (0.93, 2.45)	0.095
Phenotype 6	315	37 (11.8)	2.14 (1.25, 3.66)	0.006	2.51 (1.43, 4.04)	0.001

**Abbreviations**: AHR, hazard ratio adjusted or stratified department source, hospital origin, medical insurance type, medical history of COPD, and whether inhaled corticosteroids were used for therapy; HR, hazard ratio.

**FIGURE 2:**
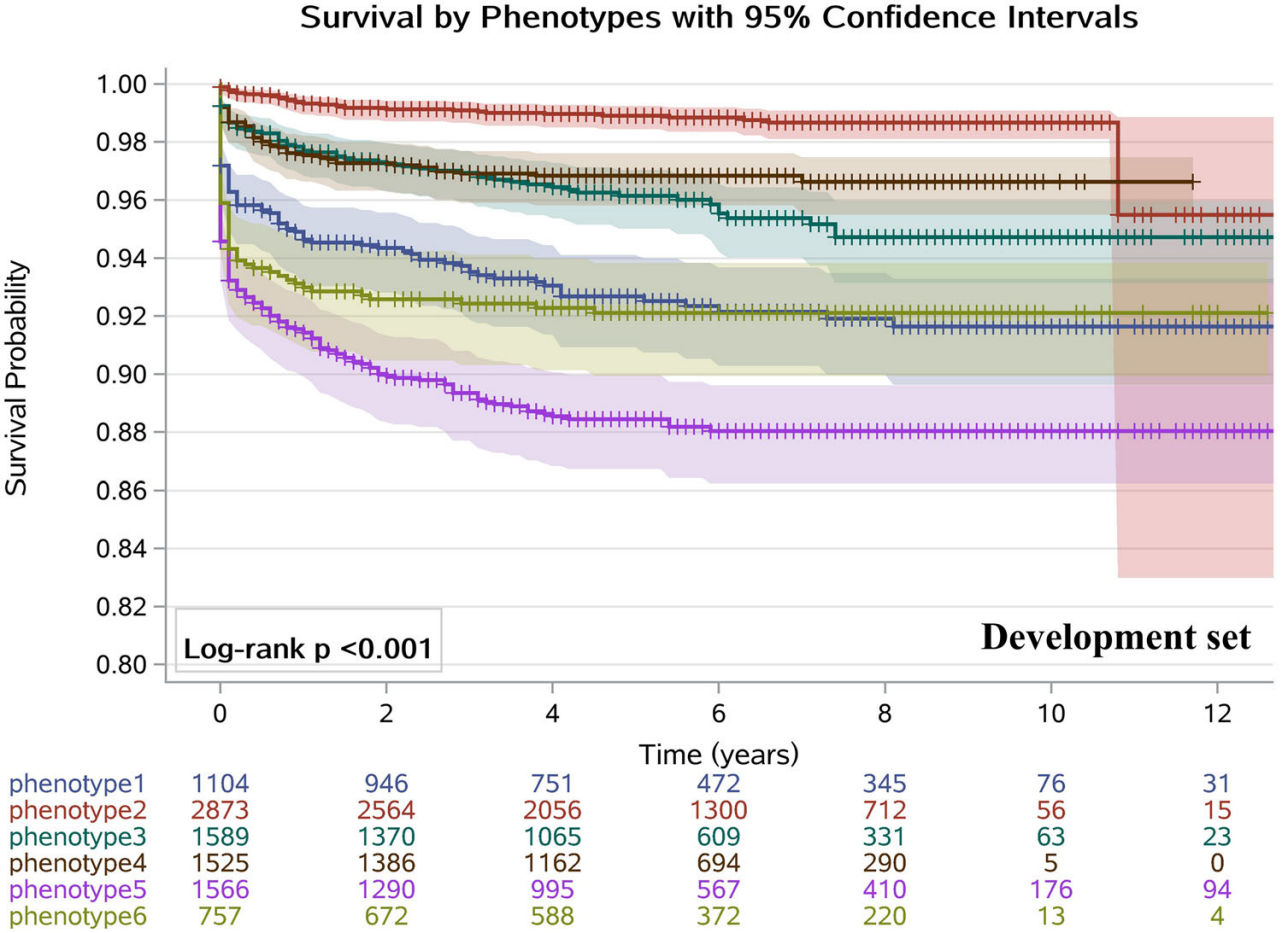
Kaplan–Meier survival curves comparing time to mortality among phenotypic subgroups in the development dataset. The plot shows estimated survival probabilities over 12 years with six groups defined by phenotypic classification. The 95% confidence interval bands for the Kaplan–Meier survival curves are shown with shaded areas. Censored observations are indicated by plus symbols (+). Numbers at risk at each 2‐year interval are shown below the plot. The log‐rank test *p*‐value (< 0.001) indicates statistically significant differences in survival distributions among phenotypic groups, suggesting phenotype is associated with differential survival outcomes.

**FIGURE 3:**
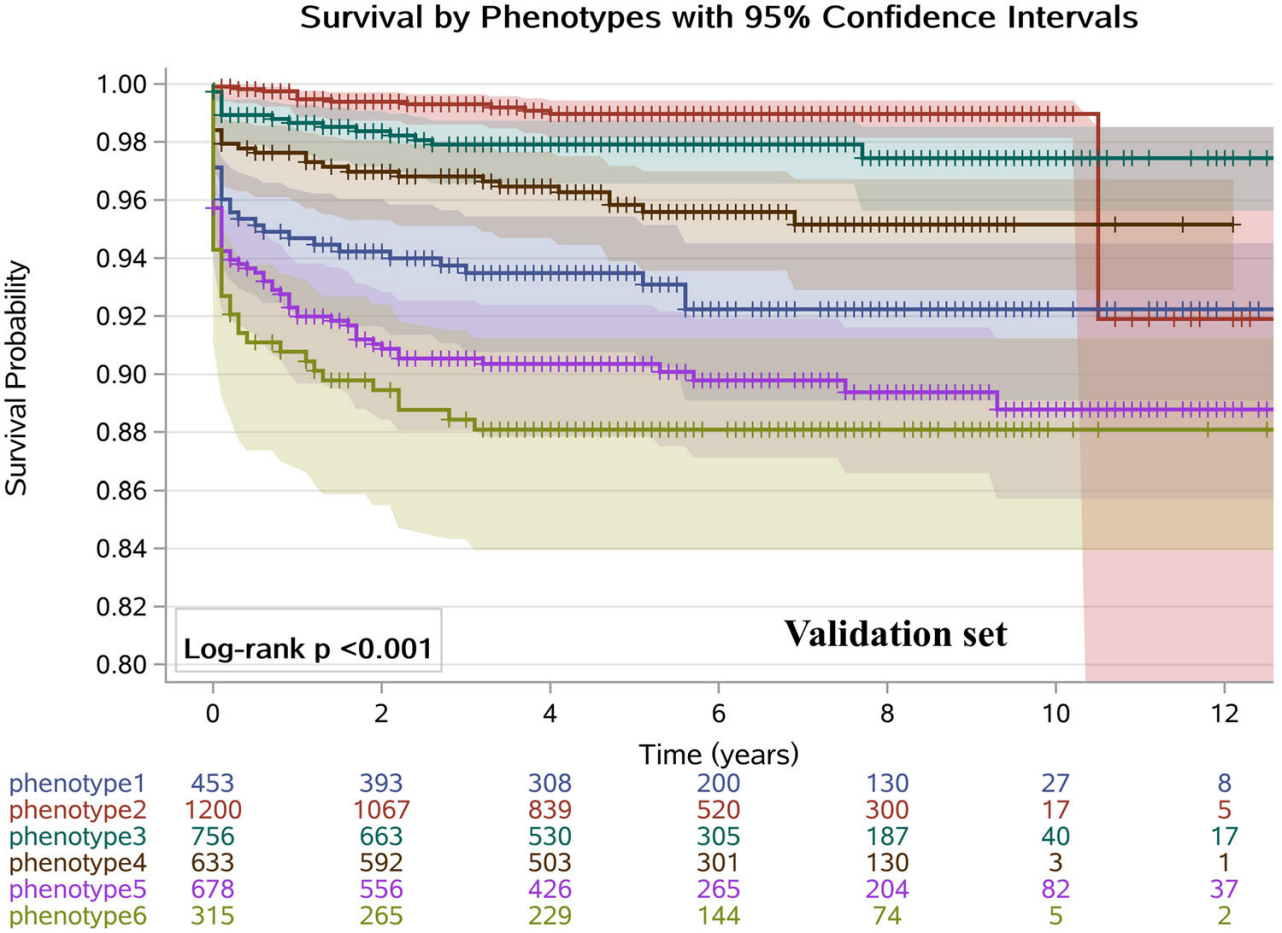
Kaplan–Meier survival curves comparing time to mortality among phenotypic subgroups in the validation dataset. The plot shows estimated survival probabilities over 12 years with six groups defined by phenotypic classification. The 95% confidence interval bands for the Kaplan–Meier survival curves are shown with shaded areas. Censored observations are indicated by plus symbols (+). Numbers at risk at each 2‐year interval are shown below the plot. The log‐rank test *p*‐value (< 0.001) indicates statistically significant differences in survival distributions among phenotypic groups, suggesting phenotype is associated with differential survival outcomes.

As shown in Figures 2 and 3, the Kaplan–Meier curves in both the development and validation datasets all clearly demonstrated that patients in Phenotype 6 had a higher risk of death compared to those in Phenotype 1 (log‐rank *p* < 0.001). The AHR of death in patients with Phenotype 2 was significantly lower than Phenotype 1 (AHR = 0.24, [95% CI: 0.16–0.36]) in the development dataset and the corresponding AHR was 0.21 (95% CI: 0.11–0.42) in the validation dataset. Similarly, compared with Phenotype 1, patients in Phenotype 3 had a reduced all‐cause mortality when controlling for other confounders in the development and validation set. A range of survival rates (88.4%–99.5%) at 1, 2, and 3 years was observed across the subgroups, with notable variations among the different phenotypes. This suggests that the six‐phenotype classification enables better stratification of all‐cause mortality risk among patients with ECOPD (Table S8).

## 3 Discussion

The early identification of ECOPD patients at risk of adverse outcomes (such as death or recurrence) is crucial for improving clinical management and facilitating precision interventions. To address this issue, this study is the first to reveal novel phenotypes of ECOPD based on large‐term periods of EHRs in mainland China. Finally, we proposed a 6‐phenotype LCA model to categorize ECOPD patients based on unique clinical characteristics. To our knowledge, we are the first to apply LCA to investigate the heterogeneity of ECOPD in a large Chinese population. To simplify the stratification of the patients, we developed and validated an LCA model that uses 12 important predictors to classify the patients into the identified phenotypes in the development set. Additionally, a SVM classifier was developed to predict the phenotypes in the validation set. The six‐class LCA model was reliable and satisfactory because it has the optimal model fit statistics and good clinical interpretation. The results suggested that patients in Phenotype 6 had the largest death risk, while Phenotype 5 was associated with a comparatively higher risk of recurrence in the development and validation dataset. Our findings have potential for early identifying ECOPD patients with higher risks of poor prognostic and providing a good example for the application of machine learning methods in disease prediction and management.

Currently, there is growing recognition that COPD is a heterogeneous disease with various underlying phenotypes [17]; therefore, the clinical and pathological phenotypes of different patients with ECOPD vary widely. Recently, many studies have applied different subtyping approaches, such as computed tomographic images [18], to interpret the heterogeneity of COPD and further investigate their relationship with disease progression [19]. A cohort study [20] showed that in comparison with non‐emphysema‐predominant COPD, the emphysema‐predominant type had a higher risk of death, which demonstrated that different COPD subtypes were associated with various clinical outcomes. Besides, Zhou and colleagues [21] proposed a clinical prediction model to assess the severity and risk of patients with ECOPD, and the overall accuracy of the proposed model was 76.2%. However, the current COPD phenotyping framework emphasizes the extent of symptoms, the time course of onset, and several biomarkers separately [22]; therefore, the present frameworks do not well disclose underlying disease mechanisms and do not aid in guiding the choice of precision therapy. Moreover, several studies have revealed some biomarkers, for instance, blood eosinophil has been extensively studied as a means of phenotyping in COPD and was associated with shorter time to next ECOPD and more subsequent ECOPD [23]. However, a relatively small sample in that study may have affected the overall validity and generalizability. A recent review paper [24] emphasized the importance of phenotyping derives from the need to identify patient clusters with common characteristics, which can guide specific treatment strategies to achieve better clinical outcomes—“right drug, right patient, right time.” In total, our study has the following novelty features compared to the current COPD phenotyping frameworks. First, to the best of our knowledge, this is the first study that provides a novel stratification of ECOPD patients based on 133 biomarkers, including blood constituents, coagulation function, liver function, renal function, exhaled breath test, demographics characteristics, comorbidities, and symptomatology features. Owing to the multi‐biomarker approach and large samples in this study, we were able to shed light on more complex pathophysiological pathways associated with COPD, which could not have been possible with single‐biomarker analysis methods in relatively small sample research. Second, we used penalized Cox models to screen multiple biomarkers of death in ECOPD patients and to identify novel phenotypes using clustering analysis; it is the first study that applies machine learning techniques to resolve heterogeneity in ECOPD using dense phenotypic data. Third, our new phenotypes can be identified at the time of hospital admission, and all patients were recruited from four tertiary hospitals in their local areas, as classified by the Chinese hospital system, ensures good regional representation across mainland China. Thus, our findings could aid in early treatment planning and enrollment of patients in experimental clinical trials. Lastly, with the use of feature importance analysis and predictive modeling, we showed that the patients can be uniquely assigned to the identified phenotypes using only 12 biomarkers, which are routinely acquired even in resource‐limited settings. This ensures that the proposed method can be made available in remote healthcare centers. Additionally, a data‐driven clustering approach may incur bias because the imputation of missing values of multiple biomarkers may affect the results of the study. To improve this potential limitation, this study derived an LCA model based on 12 key biomarkers, which are basic variables from clinical practice.

Finally, this study identified six distinct phenotypes of patients with ECOPD using a data‐driven approach, each with unique biomarker profiles and clinical characteristics. We examined the entropy value [25], a measure of separation between latent classes, to evaluate the overlap. The results (Table S3) suggest that the LCA model with six competing classes had a higher entropy (> 0.8) [26] and clear distinctions between phenotypes. These findings have important clinical implications for precision health management and risk stratification in ECOPD: Phenotype 1 with elevated Dbil and LDH levels may indicate hepatic stress or hemolysis, warranting liver function monitoring and investigation of comorbid conditions; Phenotype 2, characterized by lower NEUT_pct and higher LYMPH_pct, may indicate less severe systemic inflammation and could potentially benefit from tailored immunomodulatory therapies [27]. Similarly, a previous study suggested that the neutrophil‐to‐lymphocyte ratio is a marker that could be used to evaluate the inflammatory status of patients with COPD and predict the remission of the inflammatory process during exacerbations [28]; the coexistence of CVD and lower neutrophilic inflammation in Phenotype 3 suggests that it may be driven by cardiac dysfunction [29] rather than pure airway inflammation. Phenotype 4, characterized by a high NEUT_pct and low LYMPH_pct, suggests neutrophil‐dominated inflammation, possibly responding better to anti‐inflammatory treatments; Phenotype 5 features older patients with heart disease (gCVD), elevated liver/stress markers (Dbil/LDH), high neutrophil levels (NEUT_pct), and past surgical trauma. This profile suggests a frailty phenotype involving multiple systems and highlights a high‐risk subgroup requiring integrated cardiopulmonary, hepatic, and geriatric management. Phenotype 6 (higher rates of respiratory failure and elevated pulse) had the highest all‐cause mortality risk (AHR = 2.06) compared to Phenotype 1, suggesting these patients require urgent, aggressive intervention. This phenotyping approach enables risk‐adapted management, helping clinicians prioritize high‐risk patients and tailor therapies based on the underlying pathophysiology (inflammatory, cardiovascular, or respiratory profiles). Implementing such stratification in clinical practice could improve ECOPD outcomes using precision medicine. We further assessed the shared characteristics across classes and found that the key discriminators maintained a strong separation. Despite some shared features, each phenotype was dominated by a unique combination of traits (e.g., “Phenotype 3: Low Neutrophils percentage with gCVD” vs. “Phenotype 6: High Neutrophils percentage with gCVD”). Given that partial overlap may reflect shared pathophysiology or transitional states, which has significant clinical implications, a longitudinal study is warranted to comprehensively investigate this issue and track whether overlapping cases evolve into distinct subgroups over time.

Many patients with COPD may have several chronic comorbidities [30], such as ischemic heart disease, heart failure, diabetes, and metabolic syndrome during the progression of COPD [31]. A prospective population study [8] showed that the number and type of comorbidities among patients with COPD are associated with the risk of future death. Consistent with previous studies, we found that patients with ECOPD in Phenotype 6 were associated with a higher risk of death compared to those in other phenotypes, which may be attributable to the higher prevalence of respiratory failure, pulmonary heart disease, and gCVD. Respiratory failure is a common complication of COPD and has been frequently associated with severe ECOPD as well as higher mortality rates [32]. Cardiovascular diseases (CVDs), including pulmonary heart disease and gCVD, are frequently observed in individuals with COPD and underlying CVDs increase the severity of acute exacerbations [7]. The coexistence of these two conditions is linked to a heightened likelihood of hospitalization, extended duration of hospital stays, as well as increased mortality rates related to both all causes and CVDs [33]. Similarly, our findings demonstrated that patients with ECOPD with the above comorbidities had a higher risk of death and recurrence.

This study reported that LDH is a significant risk factor for predicting novel phenotypes that are positively associated with a higher risk of death and recurrence of ECOPD. Similarly, a recent meta‐analysis [34] suggested that increased LDH levels were strongly associated with acute respiratory distress syndrome diagnosis. LDH is an enzyme that plays a crucial role in anaerobic metabolism. It is involved in processes related to hypoxia, inflammation, and cellular damage. Therefore, LDH is a potential biomarker for assessing COPD progression. A previous study [35] conducted in primary care practices found that among patients with normal‐range bilirubin levels, relatively higher levels of bilirubin were associated with a lower risk of respiratory disease and all‐cause mortality; however, our findings suggested that Dbil is a representative biomarker of Phenotype 1 and Phenotype 5, which increases the risk of death in patients with ECOPD. A possible explanation for this discrepancy is the markedly lower median bilirubin levels in our cohort (Phenotype 1: 2.8 µmol/L [IQR, 2.7–4.0]; Phenotype 5: 2.8 µmol/L [IQR, 2.6–4.1]) relative to the previous study. Moreover, a study reported that a low LYMPH_pct was associated with disease severity and nutritional status in patients with COPD [36]. Our findings also confirmed that LYMPH_pct can be a key factor to predict the novel phenotypes in this study, and hence was postulated to be negatively related to the risk of death in patients with ECOPD. The relationship between ECOPD and LYMPH_pct can be attributed to malnutrition and systemic inflammatory burden. Similarly, our study also emphasized the importance of NEUT_pct in identifying patients with ECOPD in Phenotype 5 who had a comparatively higher risk of adverse outcomes, including death and recurrence. To our knowledge, this is the first study to confirm the association between NEUT_pct and all‐cause mortality in patients with ECOPD based on a large sample size.

We established a novel phenotype system to characterize ECOPD patients, and the predictive power of the phenotypes was reliable in both the development and validation datasets. Moreover, clinical interpretation was considered when choosing the best predictors of phenotypes. These new phenotypes can be utilized to identify patients with ECOPD who are at a high risk of unfavorable clinical outcomes and to conduct hierarchical management based on routine EHR data. Moreover, according to Occam's Razor [37], the best model is the one that achieves optimal prediction performance while utilizing fewer variables; therefore, we utilized only 12 important predictors to divide ECOPD patients into different phenotypes. This simplified model has the potential to facilitate large‐scale precision health management of ECOPD, particularly in resource‐limited settings, where comprehensive data collection and analysis may be challenging.

This study had several limitations. First, EHRs data were obtained from hospitals, which may have missing patient records. However, although the predictive power may decrease to some extent, the feasibility of predicting disease risk by applying machine learning techniques to EHRs data has been confirmed in a previous study [38]. Second, some missing clinical features were imputed based on their respective subgroups, which may introduce measurement bias. Third, all samples were hospital originated; hence, our research conclusions should be further verified in community‐sourced people. Fourth, the study did not adjust for air pollution and meteorological conditions as potential exacerbation sources; further studies should be conducted to control for the confounders. Fifth, we could not directly assess exacerbation severity; however, according to the Chronic Obstructive Lung Disease (GOLD) report, patients with COPD hospitalized for exacerbations were classified as having experienced severe exacerbations [39]. Additionally, because the exacerbation severity of patients differed by department origin, we used a Cox regression model to estimate the associations of phenotypes with all‐cause mortality risks while adjusting for department origin. Overall, although we cannot obtain exacerbation severity of COPD patients, the statistical measures in this study can partly avoid the bias. Finally, dynamic changes in the test indicators may be important factors in determining the phenotype. To partially assess the impact of dynamic changes in test indicators on the model's performance, a time‐varying coefficient was incorporated into the Cox model, allowing the covariates’ effect to change dynamically over time [16]. The results indicated that the associations of NEUT_pct, Dbil, LYMPH_pct, and LDH with all‐cause mortality remained significant (*p* < 0.001), even after accounting for time‐varying effects. Therefore, the influence of dynamic changes in these test indicators on phenotypic determination appears to be limited. Another longitudinal investigation is warranted to comprehensively evaluate the impact of the dynamic trajectories of the test indicators on phenotype determination.

In conclusion, we identified and validated six distinct clinical phenotypes with significantly different prognostic outcomes. These findings are critical for advancing beyond a generalized management approach, offering a robust framework that enables clinicians to deliver precision health interventions and target care toward the most vulnerable patients with ECOPD. This stratification allows for more efficient allocation of limited healthcare resources, directing intensive monitoring and follow‐up care to subgroups with the poorest prognoses, ultimately aiming to reduce hospital readmissions and improve long‐term survival and quality of life for the COPD population.

## 4 Methods and Materials

### 4.1 Study Design and Patient's Enrollment

**FIGURE 4:**
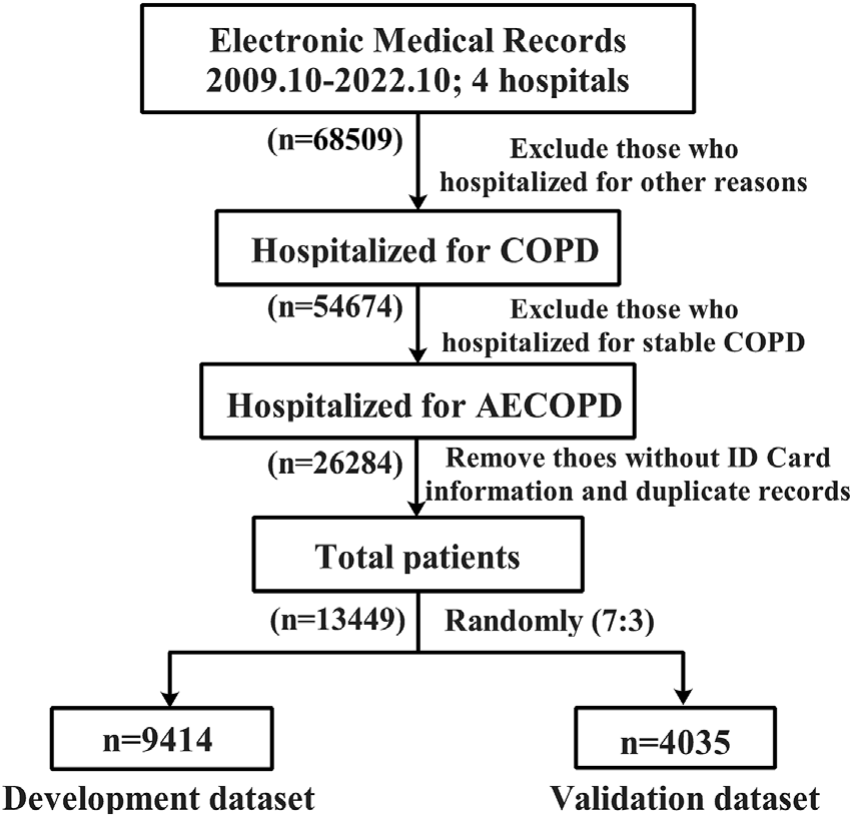
Study flow chart. The diagram illustrates the patient selection process, including inclusion and exclusion criteria, leading to the final study cohort.

This was a hospital‐based retrospective cohort study. The data analyzed in this study were downloaded from the longitudinal EMRs of patients with ECOPD enrolled in the four hospitals. The enrolled samples originated from the hospitals, with numbers of 8127, 2497, 2342, and 483, respectively. The hospital origin and department source of patients were obtained from this registry study. In this study, ECOPD was defined as an event characterized by increased dyspnea and/or cough and sputum that worsens in <14 days, which may be accompanied by tachypnea and/or tachycardia [40]. The diagnosis of ECOPD was interpreted by two experienced neurologists in the outpatient department, referenced by the Global Initiative for GOLD criteria [39] and the Rome Proposal [41]. COPD diagnosis verification procedures were defined by a visit with an International Classification of Diseases (ICD)‐10 code of J44.1 in any position or an emergency department or hospitalization with an ICD‐10 code for COPD (J44.x) in the primary position. All participants were included in the analysis if they met the following criteria: (1) patients who were diagnosed with COPD by clinicians; (2) patients who were first diagnosed with ECOPD; (3) patients who had accurate ID numbers, age, and sex; (4) patients who were hospitalized for ECOPD between October 1, 2009 and October 31, 2022. Detailed information on the study design is shown in Figure 4.

### 4.2 Clinical Information and Biomarkers

This study aimed to define the ECOPD phenotype at a specific and clinically relevant time point to minimize the confounding effect of intra‐patient variability. The index time point for the research population was defined as the time of admission. Accordingly, all baseline clinical information, along with the initial blood and exhaled breath test results obtained on the day of hospital enrollment, were included in this study. The data were stored in an EHRs database specifically designed to explore potential biomarkers of ECOPD. This database encompasses four tertiary hospitals at Guangzhou, China and contains long‐term longitudinal clinical records from 2009 to 2022, which constitute a highly representative sample for research purposes. We downloaded 133 clinical features of patients with COPD to select the most important predictive biomarkers of ECOPD (Figure S2). Demographics characteristics (age and sex) and clinical information, including medical insurance type and whether inhaled corticosteroids were used for therapy, were collected upon patient admission for ECOPD. Furthermore, the comorbidities of ECOPD (*n* = 30) were obtained from EHRs. Additionally, the first blood test and exhaled breath test results were obtained (*n* = 51). The symptomatology (*n* = 48) of patients with COPD upon admission was also stored in the EHRs database and analyzed in this study.

To address potential collinearity in the multi‐biomarker model caused by duplicate medical records and maintaining a robust sample size, we retained only the first follow‐up record for recurrent patients and excluded subsequent duplicate observations (*n* = 12,835). This approach ensured that each patient was represented by a single observation in the analyses. Consequently, all patients classified as having first COPD exacerbations were included in the final analysis (Figure 4). Furthermore, the history of COPD exacerbations documented in the registry can also be reflected in the patient's medical history of COPD. To prevent the potential confounding effect of COPD exacerbation status (new‐onset vs. relapse) on the associations between different phenotypes and prognostic risk, we adjusted for the medical history of COPD as a potential confounding factor in our subsequent association analyses. Data on the clinical symptoms of the participants were extracted from a standardized questionnaire designed for patients. The concrete indicators of comorbidities, blood tests, exhaled breath tests, and symptomatology can be found in the Supporting Information Materials.

### 4.3 Outcome Definition and Features Selection

As specific causes of death were not collected in this study, all‐cause mortality and ECOPD recurrence were selected as the primary clinical outcomes; therefore, competing risks analysis to evaluate risks from other causes of death (such as cardiovascular disease or cancer) could not be performed. Distinct ECOPD refer to a recurrence of symptoms beyond 30 days after the initial exacerbation, while exacerbations that occur within 30 days of a previous exacerbation are considered relapses [42]. This study differentiated each acute exacerbation event based on whether the time from hospital discharge to the next readmission for COPD exacerbation was > 30 days. To identify the most clinically significant features of ECOPD, this study applied rigorous screening criteria, excluding (1) features with > 90% single‐value predominance and (2) features with > 35% missing data. Following this filtering process, 67 features were eliminated before the cluster analysis.

For the missing data threshold, there is no established cutoff in the literature regarding an acceptable percentage of missing data in a dataset for valid statistical inferences [43]. Statistical guidance articles have stated that bias is likely in analyses with more than 10% missingness and that if more than 40% of the data are missing in important variables, then the results should only be considered as hypothesis generating [44]. In our opinion, the 10% standard is too strict and the 40% standard is too loose. Therefore, our approach aligns with a comparable study [45] that excluded features with > 35% missing data from the cluster analysis.

LCA models are particularly sensitive to near‐zero variance predictors because these variables can distort class separation and produce unstable parameter estimates. To address this issue, we implemented a filtering threshold to exclude variables that were overwhelmingly dominated by a single value (effectively eliminating near‐zero variance predictors). Regarding the single‐value dominance threshold, the literature lacks established guidelines for acceptable cutoffs for the dominance index. We adopted the predictor removal criteria proposed by Kuhn and Johnson, which recommend considering variable exclusion when (1) the fraction of unique values over the sample size is low (say 10%) and (2) the ratio of the frequency of the most prevalent value to the frequency of the second most prevalent value is large (say around 20) [46]. When both conditions are met for predictors in LCA models vulnerable to such variables, their removal may improve model performance. Based on these principles, we established a 90% single value dominance threshold for our study.

### 4.4 Statistical Analysis

To account for potential confounding effects of age and sex on missing data patterns, patients were stratified into four subgroups based on sex (man/woman) and age (≤ 73/> 73 years). Missing values for continuous variables were then imputed using the median value from the corresponding demographic subgroup [45]. As demonstrated in Table S9, comparative analysis revealed no significant differences between pre‐imputation and post‐subgroup‐imputation values (all *p* > 0.05), confirming the reliability of this subgroup‐based imputation approach. This stratified methodology ensured more precise missing data handling while maintaining the statistical integrity of the dataset. To further validate our approach, we conducted multiple imputation as an alternative method for handling missing data and compared the results with the subgroup‐based imputation. As shown in Table S9, the outcomes were highly consistent between the subgroup imputation and multiple imputation approaches. Notably, while most variables showed comparable results between methods, several parameters (marked with asterisks in Table S9) demonstrated significant differences between the pre‐imputation data and multiple imputation results. These findings suggest that the subgroup‐based imputation method provides greater robustness than multiple imputation for this particular dataset, as it maintained better consistency with the original data distribution.

The continuous variables were expressed as median with interquartile range if they met a skewed distribution, whereas the variables were described as mean with standard deviation if not. The difference between different phenotypes for continuous variables was determined by the Mann–Whitney *U* test. The categories variables were represented as the frequency with proportion and the comparison between different phenotypes was conducted by the chi‐square test.

In the development dataset, to select the most significant features based on their importance in the prediction of ECOPD mortality, elastic‐net penalized Cox models were used to select significant features for the next clustering analysis. Then, an unsupervised LCA was conducted to identify phenotypes that showed different clinical characteristics. We calculated AIC, CAIC, BIC, SSA‐BIC, and entropy to determine the number of phenotypes that can optimally describe the development data. The lower AIC, CAIC, BIC, and SSA‐BIC, the better clustering performance. In addition, the higher levels of entropy were associated with good model performance. Furthermore, BLRT and LMR tests were performed to compare the fit statistics between *n*‐class and (*n*‐1)‐class models. Moreover, an SVM classifier was developed to predict phenotypes using the important features based on the validation dataset. The Cox regression models were utilized to investigate the associations between different phenotypes and mortality risks. Additionally, we also conducted the Schoenfeld test for each Cox model. When a covariate is measured at baseline but its effect on the outcome varies over follow‐up time, this violates the PH assumption in Cox regression. To address this, a time‐varying coefficient or stratified covariates can be incorporated into the Cox model, allowing the covariate's effect to change dynamically with time [16]. Moreover, lognormal models that allow for the use of time‐dependent variables were also developed to assess the stability of the results.

All data management and statistical analyses were conducted using SAS Studio through SAS OnDemand for Academics (SAS Institute Inc., Cary, NC, USA) and R‐Studio 4.4.1 (Copyright 2009–2019 RStudio, Inc.) as well as Python 3.8 (Copyright 2001–2022, Python Software Foundation). A two‐side *p* value less than 0.05 was considered statistically significant.

### 4.5 Role of Funders

The funding sources listed in the manuscript did not have a role in the study design, analysis, interpretation, or writing of the manuscript. The decision to submit the manuscript for publication was made solely by the authors listed.

## Author Contributions

**Xiangqing Hou**: conceptualization, data curation, software, formal analysis, investigation, methodology, validation, visualization, funding acquisition, and writing original draft. **Zhishan Deng**: data curation, formal analysis, investigation, methodology, validation, visualization, writing – review and editing, and writing original draft. **Yumin Zhou**: investigation, methodology, validation, visualization, funding acquisition, and writing original draft. **Jie Hong**: conceptualization, investigation, and validation. **Fan Wu**: data curation, methodology, investigation, and validation. **Yuemao Li**: data curation, methodology, investigation, and validation. **Jinrong Huang**: data curation, methodology, investigation, and validation. **Cuiqiong Dai**: data curation, methodology, investigation, and validation. **Lifei Lu**: data curation, methodology, investigation, and validation. **Gaoying Tang**: data curation, methodology, investigation, and validation. **Qi Wan**: data curation, methodology, investigation, and validation. **Kunning Zhou**: data curation, methodology, investigation, and validation. **Xiaohui Wu**: investigation and validation. **Jieqi Peng**: investigation and validation. **Leqing Zhu**: investigation and validation. Ximo Chen: supervision, writing – review and editing, writing original draft, conceptualization. **Pixin Ran**: project administration, resources, supervision, writing – review and editing, writing original draft, conceptualization, and funding acquisition. All authors have read and approved the final manuscript.

## Ethics Statement

Written informed consent was obtained from all participants. This study has been approved by the Ethics Committee of the First Affiliated Hospital of Guangzhou Medical University (Reference number: 202207002).

## Conflicts of Interest

The authors declare no conflicts of interest.

## Supporting information




**Table S1**: Baseline Characteristics of Study Population.
**Table S2**: Variance Inflation Factors of the Selected 12 predictors in the LCA model based on the Development Set.
**Table S3**: Fit statistics of Latent Class Analysis with various classes based on the Subgroup Imputation Dataset.
**Table S4**: Fit Statistics for Latent Class Analysis with Various Classes based on the Multiple Imputation Dataset.
**Table S5**: The Comparison of Phenotype Characteristics in the Development set.
**Table S6**: The Schoenfeld Test results for COX Regression Model.
**Table S7**: Prognostic Outcomes of ECOPD by Phenotypes based on *Lognormal* model.
**Table S8**: The Survival Rates at one, two, and three years for each Subgroup in the Development and Validation datasets.
**Table S9**: The Comparison for Missing Variables among Imputed Before, Subgroup Imputation and Multiple Imputation.
**Figure S1**: The Distribution and Probability Plot for Age. Figures shown a box plot, and a normal probability plot as well as Quantile‐Quantile (Q‐Q) plot for the age.
**Figure S2**: Model Construction Flowchart. The diagram summarizes the feature selection process, including initial candidate variables, statistical filtering, and final model development and validation.
**Figure S3**: Cubic Spline Curves with four knots illustrate the association between Direct Bilirubin levels and Mortality. The red dashed line denotes the reference value, while the blue dashed lines represent the 95% confidence interval.
**Figure S4**: Cubic Spline Curves with four knots illustrate the Association between the Percentage of Lymphocyte and Mortality. The red dashed line denotes the reference value, while the blue dashed lines represent the 95% confidence interval.
**Figure S5**: Cubic Spline Curves with four knots illustrate the Association between Lactate dehydrogenase and Mortality. The red dashed line denotes the reference value, while the blue dashed lines represent the 95% confidence interval.
**Figure S6**: Cubic spline curves with four knots illustrate the association between age and mortality. The red dashed line denotes the reference value, while the blue dashed lines represent the 95% confidence interval.
**Figure S7**: Cubic spline curves with four knots illustrate the association between the percentage of neutrophils and mortality. The red dashed line denotes the reference value, while the blue dashed lines represent the 95% confidence interval.

## Data Availability

The datasets used and/or analyzed during the current study are available from the corresponding author on reasonable request.
