# Supplementary material for: Latent Class Analysis to Identify Novel Phenotypes in Exacerbations of COPD: A Retrospective, Multicenter Cohort Study
Source: MedComm (2020). 2025 Oct 28;6(11):e70444. doi: 10.1002/mco2.70444 (PMC12559894; doi:10.1002/mco2.70444)
Supplement: Supplementary file 1 — Table S1: Baseline Characteristics of Study Population. Table S2: Variance Inflation Factors of the Selected 12 predictors in the LCA model based on the Development Set. Table S3: Fit statistics of Latent Class Analysis with various classes based on the Subgroup Imputation Dataset. Table S4: Fit Statistics for Latent Class Analysis with Various Classes based on the Multiple Imputation Dataset. Table S5: The Comparison of Phenotype Characteristics in the Development set. Table S6: The Schoenfeld Test results for COX Regression Model. Table S7: Prognostic Outcomes of ECOPD by Phenotypes based on Lognormal model. Table S8: The Survival Rates at one, two, and three years for each Subgroup in the Development and Validation datasets. Table S9: The Comparison for Missing Variables among Imputed Before, Subgroup Imputation and Multiple Imputation. Figure S1: The Distribution and Probability Plot for Age. Figures shown a box plot, and a normal probability plot as well as Quantile‐Quantile (Q‐Q) plot for the age. Figure S2: Model Construction Flowchart. The diagram summarizes the feature selection process, including initial candidate variables, statistical filtering, and final model development and validation. Figure S3: Cubic Spline Curves with four knots illustrate the association between Direct Bilirubin levels and Mortality. The red dashed line denotes the reference value, while the blue dashed lines represent the 95% confidence interval. Figure S4: Cubic Spline Curves with four knots illustrate the Association between the Percentage of Lymphocyte and Mortality. The red dashed line denotes the reference value, while the blue dashed lines represent the 95% confidence interval. Figure S5: Cubic Spline Curves with four knots illustrate the Association between Lactate dehydrogenase and Mortality. The red dashed line denotes the reference value, while the blue dashed lines represent the 95% confidence interval. Figure S6: Cubic spline curves with four knots illustrate the asso [file MCO2-6-e70444-s001.docx]

**List of Supplementary Files**

**Table S1** Baseline Characteristics of Study Population

**Table S2** Variance Inflation Factors of the Selected 12 predictors in the LCA model based on the Development Set.

**Table S3** Fit statistics of Latent Class Analysis with various classes based on the Subgroup Imputation Dataset.

**Table S4** Fit Statistics for Latent Class Analysis with Various Classes based on the Multiple Imputation Dataset.

**Table S5** The Comparison of Phenotype Characteristics in the Development set

**Table S6** The Schoenfeld Test results for COX Regression Model

**Table S7** Prognostic Outcomes of ECOPD by Phenotypes based on *Lognormal* model

**Table S8** The Survival Rates at one, two, and three years for each Subgroup in the Development and Validation datasets

**Table S9** The Comparison for Missing Variables among Imputed Before, Subgroup Imputation and Multiple Imputation

**Figure S1** The Distribution and Probability Plot for Age. Figures shown a box plot, and a normal probability plot as well as Quantile-Quantile (Q-Q) plot for the age.

**Figure S2** Model Construction Flowchart. The diagram summarizes the feature selection process, including initial candidate variables, statistical filtering, and final model development and validation.

**Figure S3** Cubic Spline Curves with four knots illustrate the association between Direct Bilirubin levels and Mortality. The red dashed line denotes the reference value, while the blue dashed lines represent the 95% confidence interval.

**Figure S4** Cubic Spline Curves with four knots illustrate the Association between the Percentage of Lymphocyte and Mortality. The red dashed line denotes the reference value, while the blue dashed lines represent the 95% confidence interval.

**Figure S5** Cubic Spline Curves with four knots illustrate the Association between Lactate dehydrogenase and Mortality. The red dashed line denotes the reference value, while the blue dashed lines represent the 95% confidence interval.

**Figure S6** Cubic spline curves with four knots illustrate the association between age and mortality. The red dashed line denotes the reference value, while the blue dashed lines represent the 95% confidence interval.

**Figure S7** Cubic spline curves with four knots illustrate the association between the percentage of neutrophils and mortality. The red dashed line denotes the reference value, while the blue dashed lines represent the 95% confidence interval.

**Table S1 Baseline Characteristics of study population**

| **Variables** | **Values** |
| --- | --- |
| N | 13449 |
| Age, years | 73(65, 80) |
| Sex (Man), n (%) | 11210(83.4) |
| Hospital Origin, n (%) |  |
| The First Affiliated Hospital of Guangzhou Medical University | 8127(60.4) |
| The Second Affiliated Hospital of Guangzhou Medical University | 2497(18.6) |
| The Third Affiliated Hospital of Guangzhou Medical University | 2342(17.4) |
| The Five Affiliated Hospital of Guangzhou Medical University | 483(3.6) |
| Department Source, n (%) |  |
| Respiratory department | 8661(64.4) |
| ICU | 2010(15.0) |
| Others | 2778(20.6) |
| Resident Medical Insurance |  |
| Yes | 9529(70.9) |
| No | 3920(29.1) |
| Inhaled Corticosteroids for Therapy |  |
| Yes | 7529(56.0) |
| No | 5920(44.0) |

**Note:** ICU, Intensive care units.

**Table S2 Variance Inflation Factors of the Selected 12 predictors in the LCA model based on the Development Set.**

| **Variable** | **Variance inflation factor** |
| --- | --- |
| Intercept | 0 |
| Age | 1.18085 |
| Respiratory Failure | 1.13840 |
| Pulse at admission | 1.09313 |
| History of Surgical Trauma | 1.15188 |
| LYMPH_pct | 3.04867 |
| Pulmonary Heart Disease | 1.48861 |
| Dbil | 1.05707 |
| Cardiac Insufficiency | 1.24701 |
| LDH | 1.04979 |
| NEUT_pct | 2.88794 |
| Dizziness | 1.05688 |
| Generalized Cardiovascular Disease | 1.71071 |

**Note:** LYMPH_pct, the percentage of Lymphocytes; NEUT_pct, the percentage of Neutrophils; Dbil, Direct Bilirubin; LDH, lactate dehydrogenase.

**Table S3** Fit statistics of Latent Class Analysis with various classes based on the Subgroup Imputation Dataset.

| K-class | AIC | BIC | CAIC | SSA-BIC | BLRT | Adjusted LMR | Entropy |
| --- | --- | --- | --- | --- | --- | --- | --- |
|  |  |  |  |  | *P* value  (k-1 *VS* k classes) | *P* value  (k-1 *VS* k classes) |  |
| 1 | 20500.49 | 20586.29 | 20598.29 | 20548.16 |  |  | 1.00 |
| 2 | 12301.27 | 12480.02 | 12505.02 | 12400.57 | <0.001 | <0.001 | 0.82 |
| 3 | 9385.71 | 9657.41 | 9695.41 | 9536.65 | <0.001 | <0.001 | 0.88 |
| 4 | 8088.08 | 8452.72 | 8503.72 | 8290.65 | <0.001 | <0.001 | 0.84 |
| 5 | 5074.93 | 5532.53 | 5596.53 | 5329.14 | <0.001 | <0.001 | 0.85 |
| 6 | 4179.33 | 4729.88 | 4806.88 | 4485.18 | <0.001 | <0.001 | 0.81 |
| 7 | 4496.31 | 5139.81 | 5229.81 | 4853.80 | <0.001 | <0.001 | 0.78 |

**Note:** Akaike Information Criterion, AIC; the Bayesian Information Criterion, BIC; Bootstrap Likelihood Ratio Test, BLRT; Lo-Mendell-Rubin likelihood ratio, LMR.

**Table S4** Fit Statistics for Latent Class Analysis with various classes based on the Multiple Imputation Dataset.

| K-class | AIC | BIC | CAIC | SSA-BIC | BLRT | Adjusted LMR | Entropy |
| --- | --- | --- | --- | --- | --- | --- | --- |
|  |  |  |  |  | *P* value  (k-1 *VS* k classes) | *P* value  (k-1 *VS* k classes) |  |
| 1 | 18565.53 | 18651.33 | 18663.33 | 18613.20 |  |  | 1.00 |
| 2 | 11793.42 | 11972.17 | 11997.17 | 11892.72 | <0.001 | <0.001 | 0.86 |
| 3 | 7436.95 | 7708.65 | 7746.65 | 7587.89 | <0.001 | <0.001 | 0.90 |
| 4 | 4700.72 | 5065.36 | 5116.36 | 4903.29 | <0.001 | <0.001 | 0.88 |
| 5 | 4081.15 | 4538.74 | 4602.74 | 4335.36 | <0.001 | <0.001 | 0.86 |
| 6 | 3679.32 | 4229.86 | 4306.86 | 3985.17 | <0.001 | 0.050 | 0.83 |
| 7 | 3653.35 | 4296.85 | 4386.85 | 4010.84 | <0.001 | 0.648 | 0.82 |

**Note:** Akaike Information Criterion, AIC; the Bayesian Information Criterion, BIC; Bootstrap Likelihood Ratio Test, BLRT; Lo-Mendell-Rubin likelihood ratio, LMR.

**Table S5. The Comparison of Phenotype Characteristics in the Development set**

| **Variables** | **Phenotype 1**  **N=1104** | **Phenotype 2**  **N=2873** | **Phenotype 3**  **N=1589** | **Phenotype 4**  **N=1525** | **Phenotype 5**  **N=1566** | **Phenotype 6**  **N=757** | ***P*** |
| --- | --- | --- | --- | --- | --- | --- | --- |
| **Baseline Characteristics** |  |  |  |  |  |  |  |
| Age | 79.0(76.0,83.0) | 66.0(61.0,71.0) | 71.0(65.0,78.0) | 70.0(63.0,76.0) | 81.0(77.0,85.0) | 72.0(65.0,78.0) | <0.001 |
| Sex |  |  |  |  |  |  | <0.001 |
| Woman | 253(22.9) | 385(13.4) | 291(18.3) | 133(8.7) | 427(27.3) | 95(12.5) |  |
| Man | 851(77.1) | 2488(86.6) | 1298(81.7) | 1392(91.3) | 1139(72.7) | 662(87.5) |  |
| Hospital Source |  |  |  |  |  |  | <0.001 |
| 1^st^ Affiliated Hospital of GMU | 429(38.9) | 2028(70.6) | 912(57.4) | 1335(87.5) | 323(20.6) | 627(82.8) |  |
| 2^nd^ Affiliated Hospital of GMU | 353(32.0) | 505(17.6) | 255(16.0) | 83(5.4) | 532(34.0) | 49(6.5) |  |
| 3^rd^ Affiliated Hospital of GMU | 273(24.7) | 258(9.0) | 325(20.5) | 62(4.1) | 641(40.9) | 74(9.8) |  |
| 5^th^ Affiliated Hospital of GMU | 49(4.4) | 82(2.9) | 97(6.1) | 45(3.0) | 70(4.5) | 7(0.9) |  |
| Department Origin |  |  |  |  |  |  | <0.001 |
| Respiratory department | 614(55.6) | 2145(74.7) | 999(62.9) | 1158(75.9) | 592(37.8) | 552(72.9) |  |
| ICU | 201(18.2) | 235(8.2) | 291(18.3) | 65(4.3) | 502(32.1) | 90(11.9) |  |
| Others | 289(26.2) | 493(17.2) | 299(18.8) | 302(19.8) | 472(30.1) | 115(15.2) |  |
| Medical Insurance |  |  |  |  |  |  | <0.001 |
| Yes | 227(20.6) | 1011(35.2) | 512(32.2) | 425(27.9) | 307(19.6) | 221(29.2) |  |
| No | 877(79.4) | 1862(64.8) | 1077(67.8) | 1100(72.1) | 1259(80.4) | 536(70.8) |  |
| Inhaled corticosteroids for therapy |  |  |  |  |  |  | <0.001 |
| Yes | 539(48.8) | 1239(43.1) | 716(45.1) | 453(29.7) | 909(58.0) | 261(34.5) |  |
| No | 565(51.2) | 1634(56.9) | 873(54.9) | 1072(70.3) | 657(42.0) | 496(65.5) |  |
| Smoking | 668(60.5) | 2021(70.3) | 1058(66.6) | 1061(69.6) | 928(59.3) | 526(69.5) | <0.001 |
| Drinking | 191(17.3) | 592(20.6) | 308(19.4) | 294(19.3) | 232(14.8) | 133(17.6) | <0.001 |
| **Comorbidities** |  |  |  |  |  |  |  |
| Respiratory Failure | 99(9.0) | 228(7.9) | 345(21.7) | 359(23.5) | 315(20.1) | 462(61.0) | <0.001 |
| Pneumonia Lung Infection | 562(50.9) | 1139(39.6) | 668(42.0) | 750(49.2) | 766(48.9) | 387(51.1) | <0.001 |
| Bronchiectasis | 160(14.5) | 458(15.9) | 250(15.7) | 240(15.7) | 198(12.6) | 126(16.6) | 0.042 |
| Generalized Cardiovascular Disease | 85(7.7) | 95(3.3) | 1589(100.0) | 269(17.6) | 1566(100.0) | 757(100.0) | <0.001 |
| Coronary Heart Disease | 26(2.4) | 32(1.1) | 468(29.5) | 95(6.2) | 602(38.4) | 97(12.8) | <0.001 |
| Cardiac Insufficiency | 0(0.0) | 0(0.0) | 360(22.7) | 0(0.0) | 688(43.9) | 74(9.8) | <0.001 |
| Pulmonary Heart Disease | 0(0.0) | 0(0.0) | 615(38.7) | 0(0.0) | 507(32.4) | 660(87.2) | <0.001 |
| Hypertension | 482(43.7) | 783(27.3) | 710(44.7) | 497(32.6) | 945(60.3) | 246(32.5) | <0.001 |
| Diabetes | 131(11.9) | 250(8.7) | 238(15.0) | 171(11.2) | 250(16.0) | 91(12.0) | <0.001 |
| **Clinical Symptoms** |  |  |  |  |  |  |  |
| Cough Aggravate | 724(65.6) | 1907(66.4) | 1053(66.3) | 1096(71.9) | 1045(66.7) | 565(74.6) | <0.001 |
| Expectoration Aggravate | 735(66.6) | 1905(66.3) | 1033(65.0) | 1094(71.7) | 1036(66.2) | 567(74.9) | <0.001 |
| Body Temperature | 36.5(36.5,36.8) | 36.5(36.4,36.7) | 36.5(36.5,36.7) | 36.5(36.5,36.8) | 36.5(36.5,36.6) | 36.5(36.5,36.8) | <0.001 |
| Pulse at admission | 88.0(80.0,100.0) | 88.0(82.0,96.0) | 88.0(81.0,99.0) | 90.0(88.0,103.0) | 88.0(80.0,100.0) | 95.0(88.0,108.0) | <0.001 |
| Respiratory Rate | 20.0(20.0,22.0) | 20.0(20.0,21.0) | 20.0(20.0,22.0) | 20.0(20.0,22.0) | 20.0(20.0,21.0) | 22.0(20.0,23.0) | <0.001 |
| Sputum Color |  |  |  |  |  |  | <0.001 |
| White | 459(41.6) | 1191(41.5) | 611(38.5) | 595(39.0) | 615(39.3) | 290(38.3) |  |
| Yellow | 89(8.1) | 236(8.2) | 139(8.7) | 119(7.8) | 129(8.2) | 60(7.9) |  |
| White and yellow | 318(28.8) | 801(27.9) | 437(27.5) | 520(34.1) | 394(25.2) | 263(34.7) |  |
| Blood in sputum | 36(3.3) | 153(5.3) | 84(5.3) | 75(4.9) | 72(4.6) | 37(4.9) |  |
| Others | 202(18.3) | 492(17.1) | 318(20.0) | 216(14.2) | 356(22.7) | 107(14.1) |  |

Continue

| **Variables** | **Phenotype 1**  **N=1104** | **Phenotype 2**  **N=2873** | **Phenotype 3**  **N=1589** | **Phenotype 4**  **N=1525** | **Phenotype 5**  **N=1566** | **Phenotype 6**  **N=757** | ***P*** |
| --- | --- | --- | --- | --- | --- | --- | --- |
| Cough or phlegm production(≥2years) | 152(13.8) | 513(17.9) | 245(15.4) | 262(17.2) | 161(10.3) | 122(16.1) | <0.001 |
| Respite | 623(56.4) | 1648(57.4) | 899(56.6) | 922(60.5) | 922(58.9) | 471(62.2) | 0.029 |
| Respite aggravates | 413(37.4) | 1108(38.6) | 595(37.4) | 650(42.6) | 626(40.0) | 341(45.0) | 0.001 |
| Fever | 255(23.1) | 479(16.7) | 228(14.3) | 365(23.9) | 318(20.3) | 123(16.2) | <0.001 |
| Shortness of breath | 922(83.5) | 2416(84.1) | 1397(87.9) | 1388(91.0) | 1366(87.2) | 716(94.6) | <0.001 |
| Shortness of breath aggravate | 594(53.8) | 1598(55.6) | 940(59.2) | 946(62.0) | 878(56.1) | 520(68.7) | <0.001 |
| Hemoptysis | 263(23.8) | 759(26.4) | 406(25.6) | 410(26.9) | 337(21.5) | 217(28.7) | 0.001 |
| Dizziness | 465(42.1) | 822(28.6) | 620(39.0) | 382(25.0) | 845(54.0) | 168(22.2) | <0.001 |
| Chest tightness | 159(14.4) | 478(16.6) | 363(22.8) | 303(19.9) | 306(19.5) | 127(16.8) | <0.001 |
| **Medical History** |  |  |  |  |  |  |  |
| Allergy | 189(17.1) | 361(12.6) | 275(17.3) | 191(12.5) | 319(20.4) | 96(12.7) | <0.001 |
| Surgical Trauma | 452(40.9) | 453(15.8) | 474(29.8) | 63(4.1) | 989(63.2) | 23(3.0) | <0.001 |
| COPD | 695(63.0) | 1748(60.8) | 1052(66.2) | 1030(67.5) | 1009(64.4) | 573(75.7) | <0.001 |
| **Blood and exhaled breath biomarkers** |  |  |  |  |  |  |  |
| AST, U/L | 21.0(19.0,23.0) | 20.7(17.2,23.0) | 20.7(17.5,25.0) | 21.0(17.8,27.0) | 21.0(19.0,25.0) | 21.3(19.3,31.6) | <0.001 |
| Creatine kinase, U/L | 71.7(62.0,89.0) | 70.0(53.3,95.0) | 70.0(50.0,98.0) | 63.0(38.4,97.0) | 71.7(62.0,93.0) | 70.0(40.0,91.2) | <0.001 |
| Creatine kinase MB isoenzyme, U/L | 12.0(12.0,15.0) | 12.0(9.0,14.0) | 12.0(10.0,15.0) | 12.0(8.6,15.0) | 13.0(12.0,16.4) | 12.0(10.0,17.0) | <0.001 |
| LDH, U/L | 189.0(189.0,210.3) | 181.5(154.0,190.4) | 181.5(160.5,205.9) | 186.1(165.0,226.0) | 193.9(189.0,223.0) | 199.0(181.5,256.2) | <0.001 |
| Dbil, μmol/L | 2.8(2.7,4.0) | 2.3(1.7,2.8) | 2.5(2.1,3.5) | 2.2(1.6,2.9) | 2.8(2.6,4.1) | 2.7(2.0,3.6) | <0.001 |
| Bile acid, μmol/L | 4.9(3.5,5.1) | 4.8(3.7,6.5) | 4.8(3.5,6.5) | 4.9(3.8,7.6) | 4.9(3.0,4.9) | 4.9(3.9,6.9) | <0.001 |
| Tbil, μmol/L | 10.1(9.0,12.6) | 9.9(7.9,11.9) | 9.9(8.2,12.9) | 9.6(7.3,12.2) | 10.1(8.8,11.7) | 10.1(8.3,13.2) | <0.001 |
| PaCO2, mmHg | 40.5(38.4,41.3) | 42.7(38.5,44.8) | 42.7(37.7,46.5) | 42.7(37.2,50.6) | 40.5(38.3,41.3) | 49.2(40.5,64.6) | <0.001 |
| pH | 7.4(7.4,7.4) | 7.4(7.4,7.4) | 7.4(7.4,7.4) | 7.4(7.4,7.4) | 7.4(7.4,7.4) | 7.4(7.3,7.4) | <0.001 |
| PaO2, mmHg | 83.9(79.4,89.9) | 80.4(66.8,95.6) | 80.4(62.0,96.8) | 80.9(42.2,105.0) | 83.9(81.7,90.7) | 79.1(38.8,97.2) | <0.001 |
| PaO2/FiO2, mmHg | 403.0(400.5,411.5) | 389.0(358.0,437.0) | 389.0(366.0,431.0) | 391.0(336.0,484.0) | 403.0(403.0,411.5) | 402.0(305.0,466.0) | <0.001 |
| PO2, mmHg | 93.5(93.5,101.0) | 86.3(54.6,100.5) | 86.3(72.3,102.5) | 86.3(38.8,105.7) | 93.5(93.5,99.1) | 84.9(42.1,97.8) | <0.001 |
| Glucose, mmol/L | 6.7(5.9,7.1) | 6.5(5.1,7.5) | 6.5(5.3,7.5) | 7.4(6.0,9.8) | 6.7(6.2,7.5) | 6.8(6.3,8.9) | <0.001 |
| HB, g/L | 124.0(118.0,128.0) | 134.0(123.0,140.0) | 130.0(119.0,137.0) | 126.0(115.0,138.0) | 124.0(117.0,126.0) | 124.0(113.0,139.0) | <0.001 |
| RBCHB, pgh | 30.1(29.7,31.0) | 30.1(29.0,31.2) | 30.1(29.0,31.3) | 29.9(28.4,31.2) | 30.1(29.1,30.7) | 30.1(28.7,30.9) | <0.001 |
| RBCHBC, L | 329.0(325.0,333.0) | 330.0(325.0,337.0) | 330.0(322.0,335.0) | 329.0(320.0,336.0) | 329.0(323.0,331.0) | 325.0(316.0,330.0) | <0.001 |
| RBC volume, fl | 91.2(89.6,93.0) | 91.0(87.9,93.7) | 91.0(88.0,94.6) | 90.8(87.0,94.1) | 91.2(89.0,93.0) | 91.2(88.1,95.0) | <0.001 |
| MONO, 10^9^/L | 0.7(0.6,0.8) | 0.7(0.5,0.8) | 0.7(0.5,0.8) | 0.6(0.3,0.9) | 0.7(0.5,0.7) | 0.7(0.4,0.9) | 0.003 |
| MONO_pct | 8.0(6.5,8.8) | 8.1(7.1,10.5) | 8.5(7.3,10.8) | 6.1(3.2,8.5) | 8.0(6.0,8.2) | 7.2(4.2,8.7) | <0.001 |
| NEUT, 10^9^/L | 6.2(6.1,6.3) | 5.5(4.0,6.0) | 5.2(3.8,6.0) | 8.4(6.2,11.5) | 6.2(6.1,6.2) | 7.2(6.1,10.4) | <0.001 |
| NEUT_pct | 76.7(76.1,77.4) | 68.9(60.5,72.9) | 69.0(61.8,72.9) | 84.6(79.0,89.8) | 76.7(76.7,79.4) | 82.6(76.7,89.2) | <0.001 |
| RBC, 10^6^/uL | 4.2(3.9,4.4) | 4.5(4.1,4.7) | 4.4(4.0,4.7) | 4.3(3.9,4.7) | 4.2(3.9,4.3) | 4.2(3.9,4.8) | <0.001 |
| RDW, % | 14.2(13.5,14.2) | 13.9(13.2,14.3) | 13.9(13.5,14.7) | 14.0(13.4,15.0) | 14.2(13.9,14.2) | 14.4(14.0,15.6) | <0.001 |
| WBC, 10^3^/uL | 8.2(7.5,10.0) | 7.9(6.3,9.0) | 7.5(5.9,8.6) | 10.3(8.0,13.7) | 8.2(7.7,9.8) | 9.1(7.5,12.5) | <0.001 |
| BAS, 10^9^/L | 0.0(0.0,0.0) | 0.0(0.0,0.1) | 0.0(0.0,0.1) | 0.0(0.0,0.0) | 0.0(0.0,0.0) | 0.0(0.0,0.0) | <0.001 |
| BAS_pct | 0.3(0.2,0.3) | 0.4(0.3,0.6) | 0.4(0.3,0.7) | 0.2(0.1,0.4) | 0.3(0.1,0.3) | 0.3(0.1,0.4) | <0.001 |
| EOS, 10^9^/L | 0.1(0.0,0.1) | 0.2(0.1,0.3) | 0.1(0.1,0.3) | 0.0(0.0,0.1) | 0.1(0.0,0.1) | 0.0(0.0,0.1) | <0.001 |
| EOS_pct | 1.0(0.3,1.2) | 1.8(1.2,3.8) | 1.7(1.2,3.5) | 0.2(0.0,1.0) | 0.9(0.2,1.0) | 0.3(0.0,1.0) | <0.001 |
| LYMPH, 10^9^/L | 1.0(0.8,1.2) | 1.5(1.3,2.0) | 1.4(1.2,1.8) | 0.8(0.5,1.0) | 1.0(0.7,1.2) | 0.7(0.5,1.0) | <0.001 |
| LYMPH_pct | 12.9(9.3,14.6) | 20.4(15.8,27.1) | 19.8(15.8,25.5) | 7.9(5.1,10.8) | 12.9(8.3,14.4) | 8.0(4.8,12.5) | <0.001 |
| ALB, g/L | 35.0(34.2,36.3) | 36.6(35.1,39.3) | 36.6(33.8,38.5) | 35.2(31.8,38.0) | 35.0(33.4,36.1) | 34.5(31.5,36.6) | <0.001 |
| CR, μmol/L | 87.0(71.0,87.0) | 77.0(68.0,86.0) | 77.0(69.0,91.0) | 77.0(65.0,92.0) | 87.0(71.0,92.0) | 82.0(66.0,95.0) | <0.001 |
| TP, g/L | 64.4(63.2,66.7) | 64.7(62.7,69.0) | 64.7(61.6,68.5) | 64.6(59.4,68.6) | 64.4(62.4,66.0) | 63.2(57.6,65.2) | <0.001 |

**Table S6 The Schoenfeld Test results for COX Regression Model**

| **Outcomes** | **Univariate Cox Model** | ***P*** | **Multivariable Cox Model** | ***P*** |
| --- | --- | --- | --- | --- |
| **Development** |  |  |  |  |
| ECOPD Recurrence | phenotypes | 0.003 | phenotypes+ Cov1+ Cov2+ Cov3+ Cov4+ Cov5 | 0.002 |
| Mortality | phenotypes | <0.001 | phenotypes+ Cov1+ Cov2+ Cov3+ Cov4+ Cov5 | <0.001 |
| **Validation** |  |  |  |  |
| ECOPD Recurrence | phenotypes | 0.004 | phenotypes+ Cov1+ Cov2+ Cov3+ Cov4+ Cov5 | 0.002 |
| Mortality | phenotypes | 0.010 | phenotypes+ Cov1+ Cov2+ Cov3+ Cov4+ Cov5 | 0.012 |

**Note:** Cov1, department source; Cov2, hospital origin; Cov3, medical insurance type; Cov4, medical history of COPD; Cov5, whether using inhaled corticosteroids for therapy.

**Table S7 Prognostic Outcomes of ECOPD by Phenotypes based on *Lognormal* model**

| **Outcomes** | **N** | **Cases(%)** | **TR(95%CI)** | ***P*** | **'** | **ATR(95%CI)¶** | ***P*** |
| --- | --- | --- | --- | --- | --- | --- | --- |
| **Development** |  |  |  |  |  |  |  |
| *ECOPD recurrence* | |  |  |  |  |  |  |
| Phenotype 1 | 1104 | 445(40.30) | 1.00(1.00,1.00) | Ref. |  | 1.00(1.00,1.00) | Ref. |
| Phenotype 2 | 2873 | 784(27.30) | 2.71(2.17,3.39) | <0.001 |  | 2.05(1.64,2.57) | <0.001 |
| Phenotype 3 | 1589 | 509(32.00) | 1.71(1.34,2.18) | <0.001 |  | 1.52(1.19,1.95) | <0.001 |
| Phenotype 4 | 1525 | 462(30.30) | 2.10(1.63,2.69) | <0.001 |  | 1.58(1.22,2.05) | <0.001 |
| Phenotype 5 | 1566 | 591(37.70) | 1.02(0.80,1.31) | 0.850 |  | 1.12(0.88,1.42) | 0.363 |
| Phenotype 6 | 757 | 221(29.20) | 2.09(1.55,2.83) | <0.001 |  | 1.66(1.22,2.25) | 0.001 |
| *Mortality* | |  |  |  |  |  |  |
| Phenotype 1 | 1104 | 82(7.40) | 1.00(1.00,1.00) | Ref. |  | 1.00(1.00,1.00) | Ref. |
| Phenotype 2 | 2873 | 33(1.10) | 689.10(169.31,2804.43) | <0.001 |  | 196.50(49.11,786.30) | <0.001 |
| Phenotype 3 | 1589 | 64(4.00) | 10.95(3.24,37.01) | <0.001 |  | 8.67(2.53,29.68) | <0.001 |
| Phenotype 4 | 1525 | 48(3.10) | 25.29(6.93,92.28) | <0.001 |  | 7.94(2.08,30.26) | 0.002 |
| Phenotype 5 | 1566 | 178(11.40) | 0.15(0.05,0.43) | <0.001 |  | 0.35(0.12,1.00) | 0.050 |
| Phenotype 6 | 757 | 59(7.80) | 0.66(0.18,2.41) | 0.527 |  | 0.26(0.07,0.98) | 0.048 |
| **Validation** |  |  |  |  |  |  |  |
| *ECOPD recurrence* | |  |  |  |  |  |  |
| Phenotype 1 | 453 | 174(38.41) | 1.00(1.00,1.00) | Ref. |  | 1.00(1.00,1.00) | Ref. |
| Phenotype 2 | 1200 | 360(30.00) | 1.92(1.35,2.73) | <0.001 |  | 1.50(1.05,2.14) | 0.025 |
| Phenotype 3 | 756 | 229(30.29) | 1.72(1.17,2.51) | 0.005 |  | 1.55(1.06,2.26) | 0.022 |
| Phenotype 4 | 633 | 184(29.07) | 2.02(1.36,3.00) | <0.001 |  | 1.49(0.99,2.23) | 0.053 |
| Phenotype 5 | 678 | 243(35.84) | 0.99(0.67,1.45) | 0.953 |  | 1.15(0.79,1.68) | 0.470 |
| Phenotype 6 | 315 | 96(30.48) | 1.42(0.88,2.28) | 0.148 |  | 1.17(0.72,1.88) | 0.524 |
| *Mortality* | |  |  |  |  |  |  |
| Phenotype 1 | 453 | 32(7.06) | 1.00(1.00,1.00) | Ref. |  | 1.00(1.00,1.00) | Ref. |
| Phenotype 2 | 1200 | 12(1.00) | 858.10 (92.76,7938.73) | <0.001 |  | 221.70(25.29,1943.69) | <0.001 |
| Phenotype 3 | 756 | 16(2.12) | 75.56(9.54,598.58) | <0.001 |  | 65.69(8.53,505.82) | <0.001 |
| Phenotype 4 | 633 | 27(4.27) | 7.69(1.21,48.87) | 0.031 |  | 3.96(0.59,26.63) | 0.157 |
| Phenotype 5 | 678 | 68(10.03) | 0.25(0.05,1.26) | 0.093 |  | 0.50(0.10, 2.51) | 0.398 |
| Phenotype 6 | 315 | 37(11.75) | 0.11(0.02,0.73) | 0.023 |  | 0.08(0.01,0.48) | 0.009 |

**Note:** TR, Time Ratio; ATR, Time Ratio after Adjusted department source, hospital origin, medical insurance type, medical history of COPD and whether inhaled corticosteroids were used for therapy.

**Table S8 The Survival Rates at one, two, and three years for each Subgroup in the Development and Validation datasets**

| **Survival rates** | **Phenotype1** | **Phenotype2** | **Phenotype3** | **Phenotype4** | **Phenotype5** | **Phenotype6** |
| --- | --- | --- | --- | --- | --- | --- |
| Development set | |  |  |  |  |  |
| 3-year | 93.5% | 99.1% | 96.9% | 96.9% | 89.4% | 92.4% |
| 2-year | 94.3% | 99.1% | 97.2% | 97.3% | 89.9% | 92.6% |
| 1-year | 94.6% | 99.3% | 97.7% | 97.6% | 91.4% | 93.0% |
| Validation set | |  |  |  |  |  |
| 3-year | 93.5% | 99.3% | 97.9% | 96.8% | 90.5% | 88.4% |
| 2-year | 94.2% | 99.4% | 98.4% | 97.0% | 90.9% | 89.5% |
| 1-year | 94.7% | 99.5% | 98.7% | 97.6% | 92.0% | 90.8% |

**Table S9** The comparison for missing variables among imputed before, subgroup imputation and multiple imputation

| **Characteristics** | **Imputed Before** | | | **Subgroup Imputation** | **Multiple Imputation** |
| --- | --- | --- | --- | --- | --- |
|  | **Number missing** | **Percent missing,%** | **Median(p25,p75)** | **Median(p25,p75)** | **Median(p25,p75)** |
| AST, U/L | 2772 | 20.6 | 20.9(17.0, 27.0) | 20.7(18.0, 24.9) | 20.9(17.0,27.0) |
| Creatine Kinase, U/L | 2961 | 22.0 | 69.0(44.0, 112.0) | 70.0(50.0, 95.7) | 69.0(43.4,111.9) |
| Creatine Kinase MB Isoenzyme, U/L | 2988 | 22.2 | 12.0(9.0, 17.0) | 12.0(10.0, 15.0) | 12.0(9.0,17.0) |
| LDH, U/L | 2956 | 22.0 | 186.0(159.0, 224.0) | 189.0(166.0, 211.0) | 186.7(159.0,225.0) |
| Dbil, μmol/L | 2883 | 21.4 | 2.5(1.8, 3.7) | 2.6(2.0, 3.3) | 2.6(1.8,3.8) |
| Bile Acid, μmol/L | 2930 | 21.8 | 4.8(3.2, 7.3) | 4.8(3.6, 6.3) | 4.7(3.2,7.2) |
| Tbil, μmol/L | 2763 | 20.5 | 9.9(7.4, 13.3) | 9.9(8.0, 12.2) | 9.8(7.3,13.3) |
| PaCO2, mmHg | 3331 | 24.8 | 41.9(36.5, 49.7) | 41.3(38.3, 46.3) | 41.9(36.5,50.1) |
| pH | 3318 | 24.7 | 7.4(7.4, 7.4) | 7.4(7.4, 7.4) | 7.4(7.4,7.4)* |
| PaO2, mmHg | 3475 | 25.8 | 81.5(45.0, 105.5) | 81.7(66.9, 97.1) | 81.3(44.5,106.0) |
| PaO2/FiO2, mmHg | 4539 | 33.7 | 394.0(331.0, 499.0) | 403.0(362.0, 438.0) | 392.0(327.0,500.0) |
| PO2, mmHg | 3686 | 27.4 | 89.9(40.9, 107.0) | 93.5(69.5, 101.6) | 88.9(40.4,106.9) |
| Glucose, mmol/L | 2769 | 20.6 | 6.6(5.3, 8.6) | 6.5(5.5, 8.0) | 6.5(5.2,8.6) |
| HB, g/L | 2683 | 19.9 | 128.0(115.0, 140.0) | 127.0(119.0, 136.0) | 126.0(110.0,139.0)* |
| RBCHB, pgh | 2213 | 16.5 | 30.0(28.7, 31.4) | 30.1(29.0, 31.0) | 29.9(27.9,31.3)* |
| RBCHBC, L | 2214 | 16.5 | 329.0(321.0, 336.0) | 329.0(323.0, 335.0) | 328.0(319.0,336.0)* |
| RBC volume, fl | 2213 | 16.5 | 91.0(87.2, 94.6) | 91.0(88.1, 93.9) | 90.6(86.5,94.4)* |
| MONO, 10^9^/L | 2214 | 16.5 | 0.6(0.4, 0.9) | 0.7(0.5, 0.8) | 0.6(0.4,0.9)* |
| MONO_pct | 2215 | 16.5 | 7.9(5.6, 10.2) | 7.9(6.2, 9.7) | 7.8(5.6,10.1) |
| NEUT 10^9^/L | 4660 | 34.7 | 6.0(4.3, 8.8) | 6.0(4.3, 8.8) | 6.0(4.2,8.6) |
| NEUT_pct | 4660 | 34.7 | 74.5(64.4, 84.2) | 74.5(64.4, 84.2) | 73.9(64.5,82.8)* |
| RBC, 10^6^/uL | 2215 | 16.5 | 4.3(3.9, 4.7) | 4.3(3.9, 4.7) | 4.3(3.8,4.7)* |
| RDW_pct | 3379 | 25.1 | 14.0(13.2, 15.0) | 14.0(13.5, 14.6) | 14.1(13.3,15.2)* |
| WBC, 10^3^/uL | 2213 | 16.5 | 8.2(6.4, 10.9) | 8.2(6.8, 10.2) | 8.3(6.4,10.9) |
| BAS, 10^9^/L | 2213 | 16.5 | 0.0(0.0, 0.1) | 0.0(0.0, 0.0) | 0.0(0.0,0.1) |
| BAS_pct | 2849 | 21.2 | 0.3(0.1, 0.6) | 0.3(0.2, 0.5) | 0.3(0.2,0.6) |
| EOS, 10^9^/L | 2218 | 16.5 | 0.0(0.0, 0.2) | 0.1(0.0, 0.2) | 0.1(0.0,0.2)* |
| EOS_pct | 2850 | 21.2 | 1.1(0.1, 3.0) | 1.0(0.3, 2.4) | 1.3(0.2,3.0)* |
| LYMPH, 10^9^/L | 2215 | 16.5 | 1.2(0.8, 1.7) | 1.2(0.8, 1.6) | 1.2(0.8,1.7) |
| LYMPH_pct | 2213 | 16.5 | 14.7(8.5, 22.6) | 14.4(9.6, 20.8) | 14.7(8.8,22.3) |
| ALB, g/L | 2716 | 20.2 | 35.9(32.7, 38.9) | 35.6(33.7, 38.0) | 35.8(32.6,38.8)* |
| CR, μmol/L | 3442 | 25.6 | 79.0(66.0, 96.0) | 78.0(70.0, 89.0) | 79.0(66.0,96.5) |
| TP, g/L | 2789 | 20.7 | 64.8(60.3, 69.2) | 64.7(61.7, 68.0) | 64.7(60.2,69.1) |

**Note:** Subgroup Imputation indicates patients were divided into four subgroups according to their age (<=73 and >73) and gender (man and woman), and the missing values were imputed based on the median of the respective subgroups; The asterisk (*) indicates that there existed significant difference between imputed before and multiple imputation after.

***
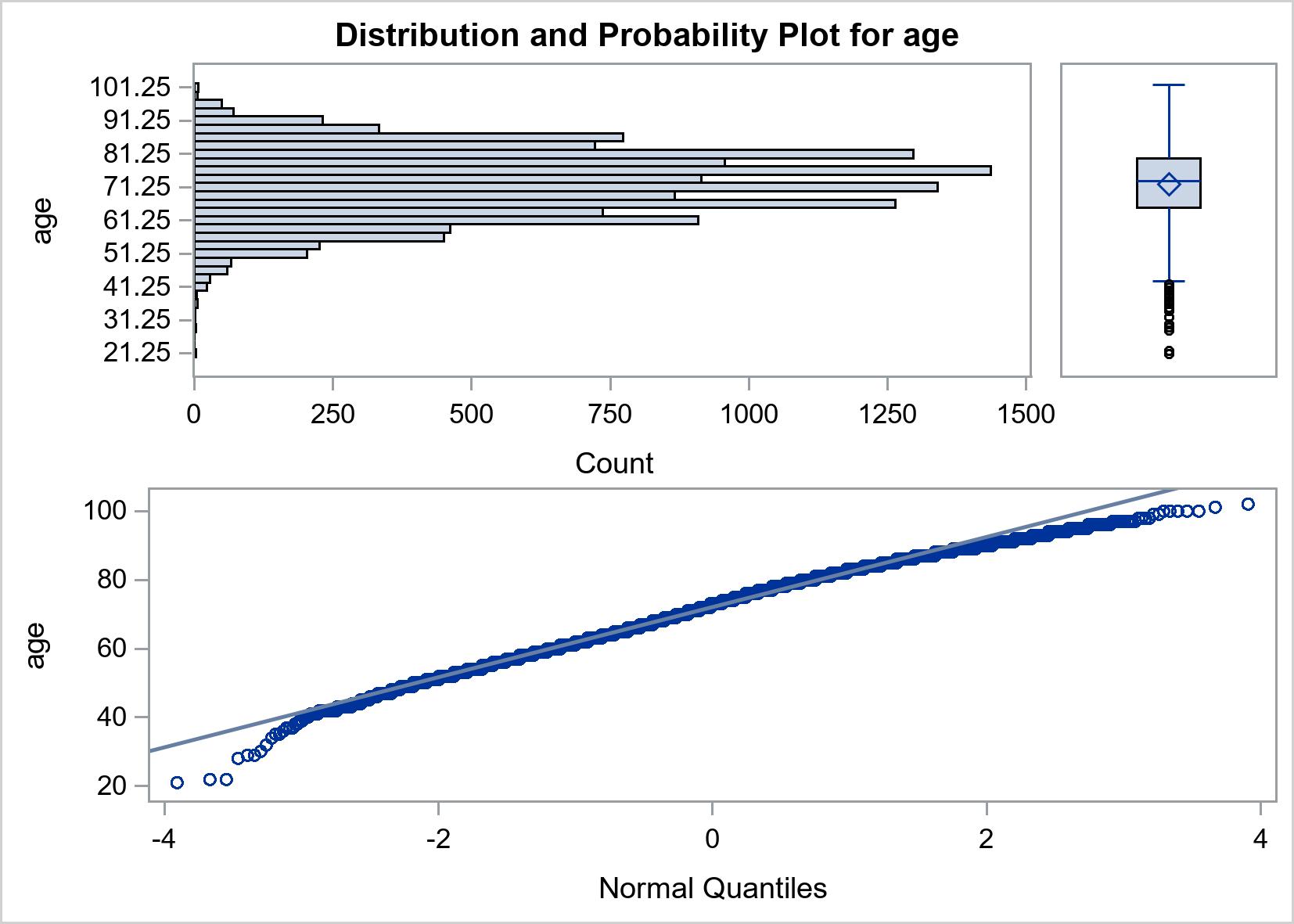
***

**Figure S1**

**
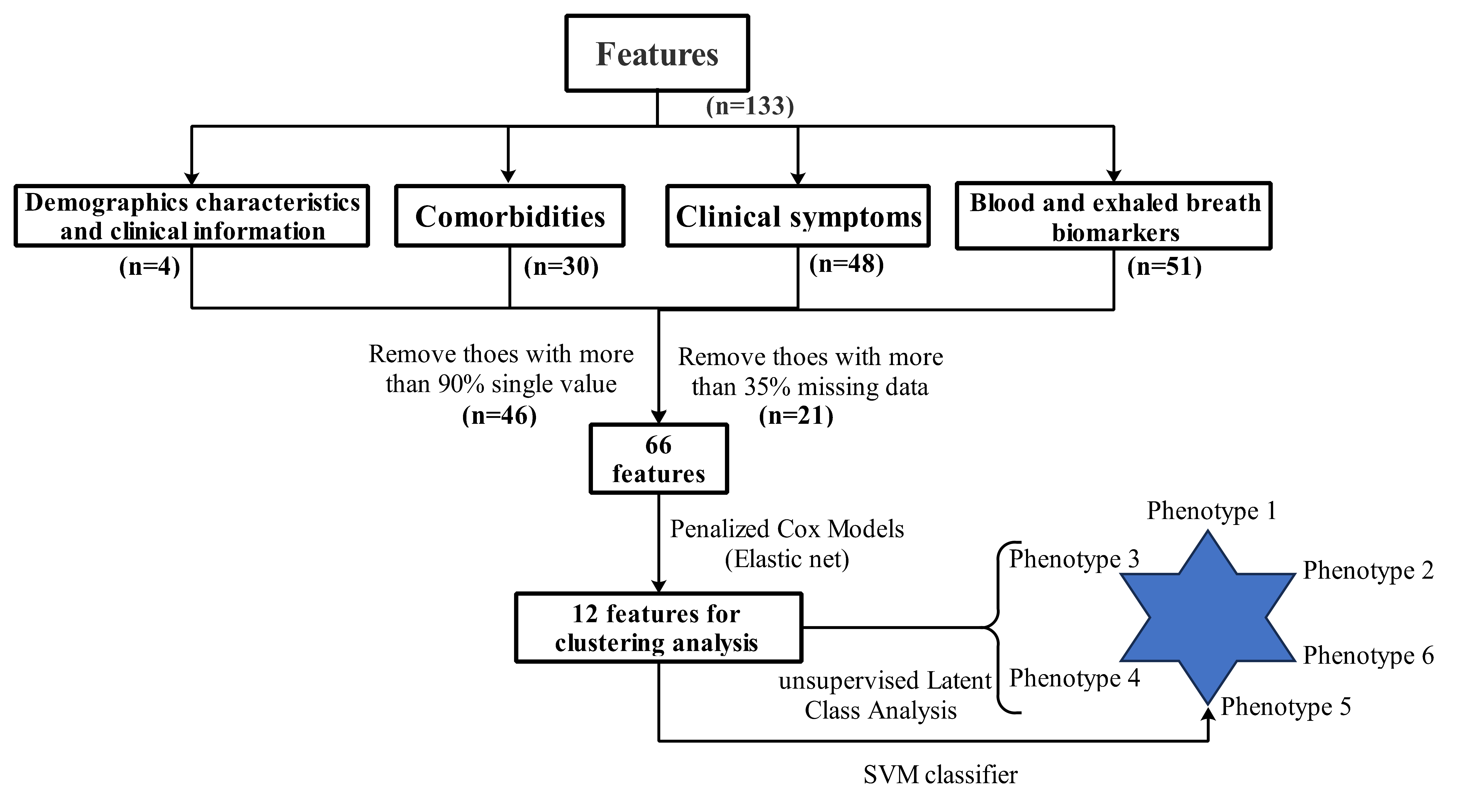
**

**Figure S2**

**
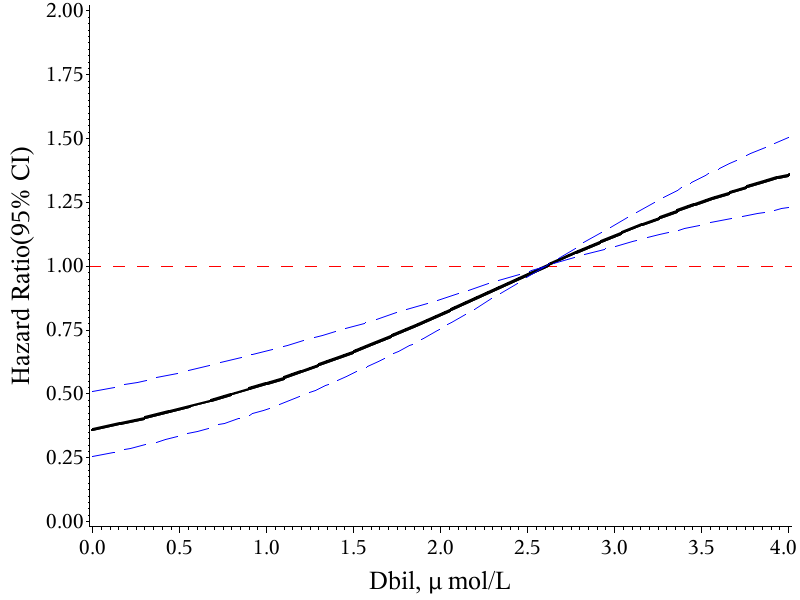
**

**Figure S3**

**
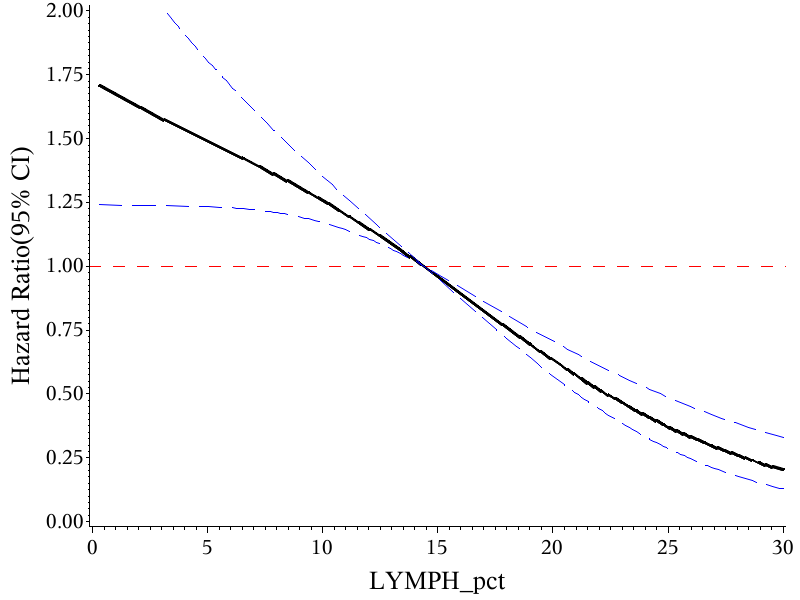
**

**Figure S4**

**
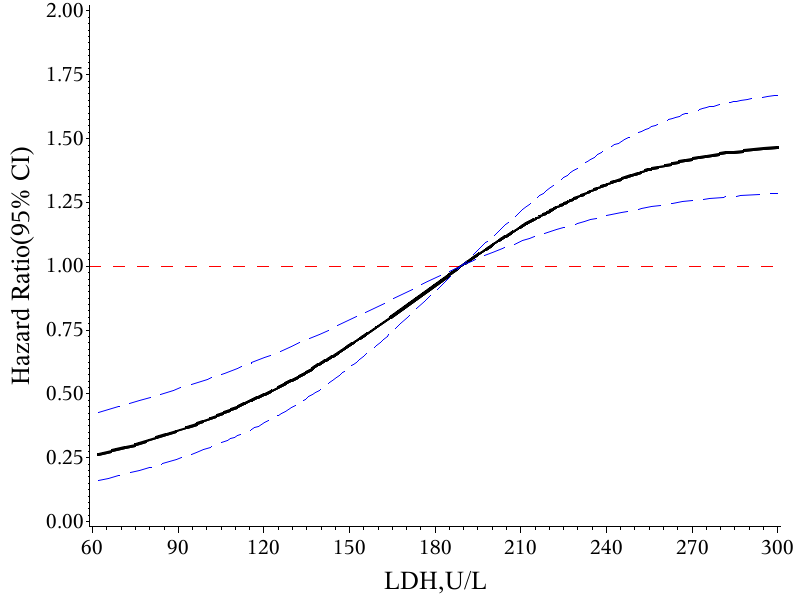
**

**Figure S5**

**
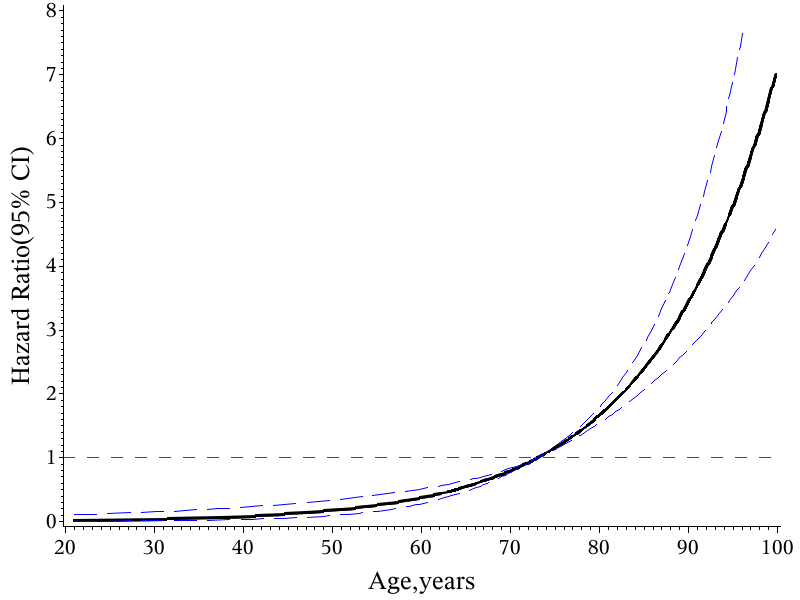
**

**Figure S6**

**
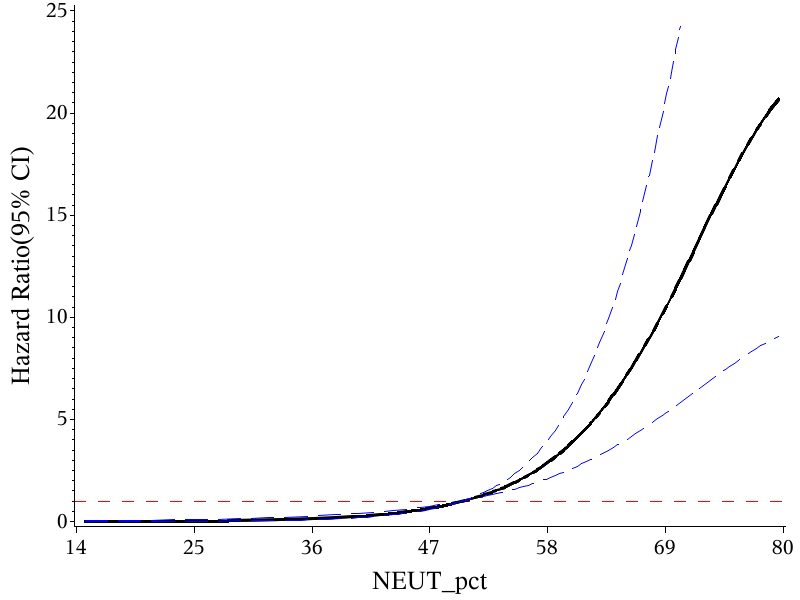
**

**Figure S7**

***The description of the biomarkers***

The comorbidities of ECOPD including respiratory failure, pneumonia lung infection, severe pneumonia, asthma, tuberculosis, bronchiectasis, interstitial lung disease, sleep apnea syndrome, pulmonary aspergillus infection, generalized cardiovascular disease, myocardial infarction angina pectoris, coronary heart disease, heart failure, cardiac insufficiency, arrhythmia, valvular heart disease, cardiomyopathy, pulmonary hypertension, pulmonary heart disease, pulmonary embolism, cerebral infarction, cerebral hemorrhage, arteriovenous thromboembolism, malignant tumor, lung malignant tumors, lung space-occupying shadow, hypertension, diabetes, osteoporosis, parasitic disease.

The first blood test covers B-type natriuretic peptide precursor, aspartate aminotransferase, high sensitivity troponin I, creatine kinase, creatine kinase MB isoenzyme, lactate dehydrogenase, myoglobin, exhaled nitric oxide levels, total cholesterol, direct bilirubin (Dbil), high-density lipoprotein (HDL), low-density lipoprotein (LDL), lipidemia index, serum total bile acids, total bilirubin (Tbil), triglycerides, partial pressure of carbon dioxide, pH, partial pressure of oxygen in arterial blood (PaO2), oxygenation index (PaO2/FiO2), arterial blood partial pressure of oxygen (PO2), respiratory index, Standard bicarbonate concentration, hemoglobin concentration (measurement), glycated hemoglobin (HbA1a), glycated hemoglobin (HbA1b), blood sugar (fasting), fasting serum insulin measurement, hemoglobin (HB), average Hb content of red blood cells (RBCHB), average red blood cell Hb concentration (RBCHBC), mean red blood cell volume (RBC volume), number of monocytes (MONO), the percentage of monocyte (MONO_pct), neutrophil count (NEUT), the percentage of neutrophil (NEUT_pct), red blood cells (RBC), red blood cell distribution width coefficient of variation (RDW), white blood cell (WBC), basophil count (BAS), the percentage of basophil(BAS_pct), eosinophil count(EOS), the percentage of eosinophil (EOS_pct), lymphocyte count(LYMPH), the percentage of lymphocyte (LYMPH_pct), albumin(ALB), urea nitrogen(UN), creatinine(CR), serum cystatin C, total protein(TP), uric acid(UA), Lactate dehydrogenase(LDH), the partial pressure of carbon dioxide(PaCO2).

The symptomatology covered cough, cough aggravate, expectoration, expectoration aggravate, type of expectoration, sputum color, cough or phlegm production for more than 2 years, respite, respite aggravate, difficulty breathing, difficulty breathing aggravate, fever, shortness of breath, shortness of breath aggravate, hemoptysis, nature of hemoptysis, dizziness, chest pain, chest tightness, headache, nausea, vomit, palpitations, cyanosis, lower extremity edema/edema, nasal congestion, runny nose, chilly, Chills/chills, weakness, muscle ache, sore throat, rash, weight change, history of allergy, history of surgical trauma, smoking, years of smoking, smoking daily amount, drinking, familial disease, family history of respiratory disease, body temperature, pulse, respiratory rate, systolic blood pressure, diastolic blood pressure, medical history of COPD.
